# Effects of Lipid Headgroups on the Mechanical Properties and In Vitro Cellular Internalization of Liposomes

**DOI:** 10.1021/acs.langmuir.4c04363

**Published:** 2025-01-21

**Authors:** Jiaming Xu, Stephen Adepoju, Simran Pandey, Jimena Pérez Tetuán, Mary Williams, Rudolf G. Abdelmessih, Debra T. Auguste, Francisco R. Hung

**Affiliations:** † Department of Chemical Engineering, Northeastern University, Boston, Massachusetts 02115, United States; ‡ Department of Bioengineering, Northeastern University, Boston, Massachusetts 02115, United States

## Abstract

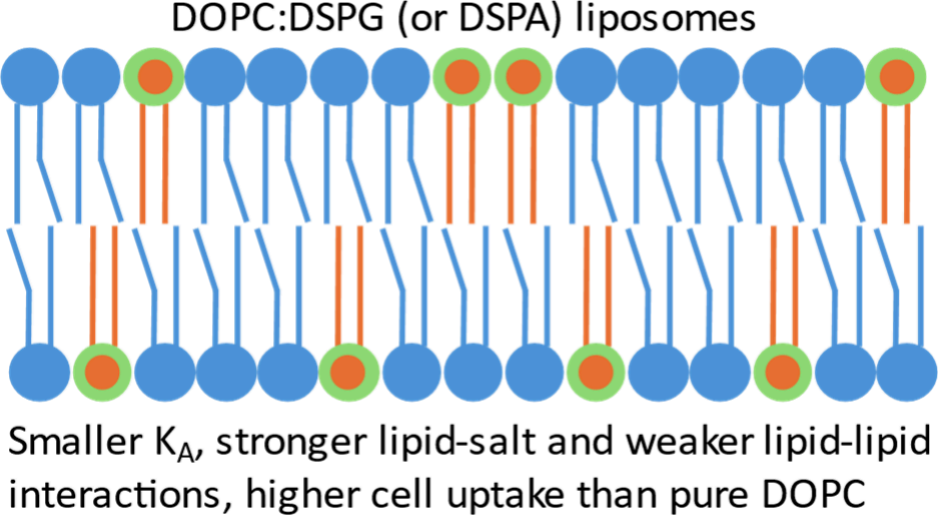

We performed all-atom and coarse-grained simulations of lipid bilayer mixtures of the unsaturated lipid DOPC, with saturated lipids having the same 18-carbon acyl tails and different headgroups, to understand their mechanical properties. The secondary lipids were DSPG, DSPA, DSPS, DSPC, and DSPE. The DOPC:DSPG system with 65:35 molar ratio was the softest, with area compressibility modulus *K*_*A*_ ∼ 22% smaller than the pure DOPC value. Raising the mole % of DOPC leads to increases in *K*_*A*_, yet at any given composition the *K*_*A*_ trend is DSPG < DSPA < DSPS < DSPC < DSPE. Lipid–lipid interactions are weaker in DOPC:DSPG mixtures and stronger in DSPE systems. The head and phosphate groups of the secondary lipids DSPG, DSPA, and DSPS interact strongly with salt ions. Adding secondary lipids leads to DOPC having more ordered acyl tails relative to pure DOPC systems. No evidence of phase separation or inhomogeneities was observed in our simulations. We synthesized three liposomal formulations, L-DOPC (pure DOPC) and L-DOPC/DSPG and L-DOPC/DSPA, both with 15 mol % of secondary lipid. L-DOPC/DSPA had approximately 3- and 2-times higher in vitro internalization by normal epithelial (EpH4-Ev) and metastatic breast cancer (4T1) cells, compared with L-DOPC. The uptake of L-DOPC/DSPG by EpH4-Ev cells was almost 2-fold compared to L-DOPC, but both liposomes had similar uptakes by cancer cells. As L-DOPC/DSPG and L-DOPC/DSPA have similar *K*_*A*_ values, we presumed that the mechanical properties, possibly in combination with the higher negative surface charges in L-DOPC/DSPA and differences in effective liposome diameters and diffusivities, contributed to these observations.

## Introduction

Liposomes are spherical vesicles formed by a lipid bilayer encapsulating a central core that can protect and carry active ingredients with applications in the pharmaceutical, nutraceutical, cosmetic, food, and textile industries.^1^ For example, they have been used to encapsulate additives, antimicrobials, flavor agents, preservatives, nutrients, and active cosmetic ingredients, to protect them against degradation by enzymes and chemicals,^2,3^ and to improve skin absorption of cosmetics.^4,5^ Liposomes also have applications in biosensing of pesticides,^6,7^ in cleaning of oil spills,^8,9^ in textile processing as detergents and dye solubilizers,^10^ and in biofuels based on lipid droplet technologies.^11−13^

Liposomes and lipid nanoparticles (LNPs) are widely recognized as promising drug delivery vehicles due to their stability, bioavailability, and low toxicity, among other advantages. However, in applications such as cancer treatment, the challenge of improving the efficacy of delivery of drugs carried by nanoparticles (NPs) to tumors remains a central topic of interest. As NPs journey from their injection site to tumors, blood proteins immediately adsorb onto their surface as they are marked for elimination by the body’s immune system. The NPs that reach tumor sites first need to leak out of the bloodstream, then penetrate the solid tumor and be internalized by tumor cells to finally deliver their cargo drugs. As a result, on average only 0.7% of injected NPs reach tumor sites.^14−16^ Research on improving NPs in drug delivery has mainly focused on their size, shape, and surface chemistry; only recently the significant role that NP mechanics can play has been studied.^14,17^ Compared to more rigid NPs, soft NPs seem to have longer circulation times in blood and less accumulation in organs such as the spleen, which filters foreign substances from blood. In some studies, more deformable NPs seem to penetrate tumors better,^18,19^ but in other studies NPs with intermediate elasticity seem to have larger tumor accumulations compared to softer and stiffer counterparts, which were theorized as deforming too much or too little.^20^ Likewise, some studies report that soft NPs require more energy by cancer cells to uptake them as they deform during internalization.^21^ A recent study^19^ indicates that softer NPs can be internalized by cancer cells via membrane fusion and endocytosis, whereas rigid NPs can only undergo endocytosis and thus have lower uptake by cancer cells. These apparent inconsistencies in results were attributed^14^ to limited understanding of the processes that NPs undergo in their journey from injection to tumor sites, and to the different experimental settings among different studies (e.g., in vitro vs in vivo, different experimental techniques). Furthermore, the studies above have used different types of NPs (silica nanocapsules, hydrogel NPs, lipid and hybrid polymeric-lipid NPs), as no single NP type would allow access to the whole range of possible elasticity values (as measured by the Young’s modulus, which for NPs can span 7 orders of magnitude, ∼1 kPa to ∼10^7^ kPa).^14^ Overall, these factors point to a poor fundamental understanding of how to tune the mechanical properties of NPs.

Elastic liposomes and LNPs could allow them to reversibly deform and penetrate through fibrotic tissue present in cancer.^19,20,22^ Fibrosis in cancer is caused by the abnormal activation of stromal cells such as fibroblasts and myofibroblasts. These cells prolifically synthesize copious quantities of extracellular matrix (ECM) proteins, including collagen and fibronectin, forming fibrous tissue.^23,24^ ECM proteins can alter the physical and mechanical properties of the tumor microenvironment, suppressing immune activity^25,26^ and making it more difficult for drugs to diffuse through fibrous tissue.^27,28^ 1,2-Dioleoyl-*sn*-glycero-3-phosphocholine (DOPC), a phospholipid with acyl tails of 18 carbon atoms and a single double bond between carbons 9 and 10 in each of their tails (Table 1), is an ideal ingredient for lipid nanoparticles because of its biocompatibility, low melting point, stability, hydrophobic properties, and versatility. Pure DOPC liposomes have a Young’s moduli of 45 kPa,^14,19^ and in practice are among the most elastic type of liposomes. Previous experimental and simulation results^29^ have shown that lipid bilayers formed by phospholipids with multiple double bonds in their carbon tails (e.g., DLiPC, PUPC, DNPC from ref (29)), have bending moduli *k*_*C*_ that are smaller than the DOPC value and thus are less rigid. However, in practice, the multiple double bonds make the resulting liposomes more prone to oxidation, less stable, and more susceptible to leak their cargo in the long term, making them impractical for the applications mentioned above. Further, DOPC is zwitterionic, has an area per lipid of 66.3 Å^2^, and is in liquid phase at 37 °C as its melting temperature is −17 °C.^30^ These properties make DOPC a suitable component for delivering drugs and other bioactive molecules to target cells and tissues and for creating LNPs with different properties such as size, surface charge, and stability.

**Table 1:**
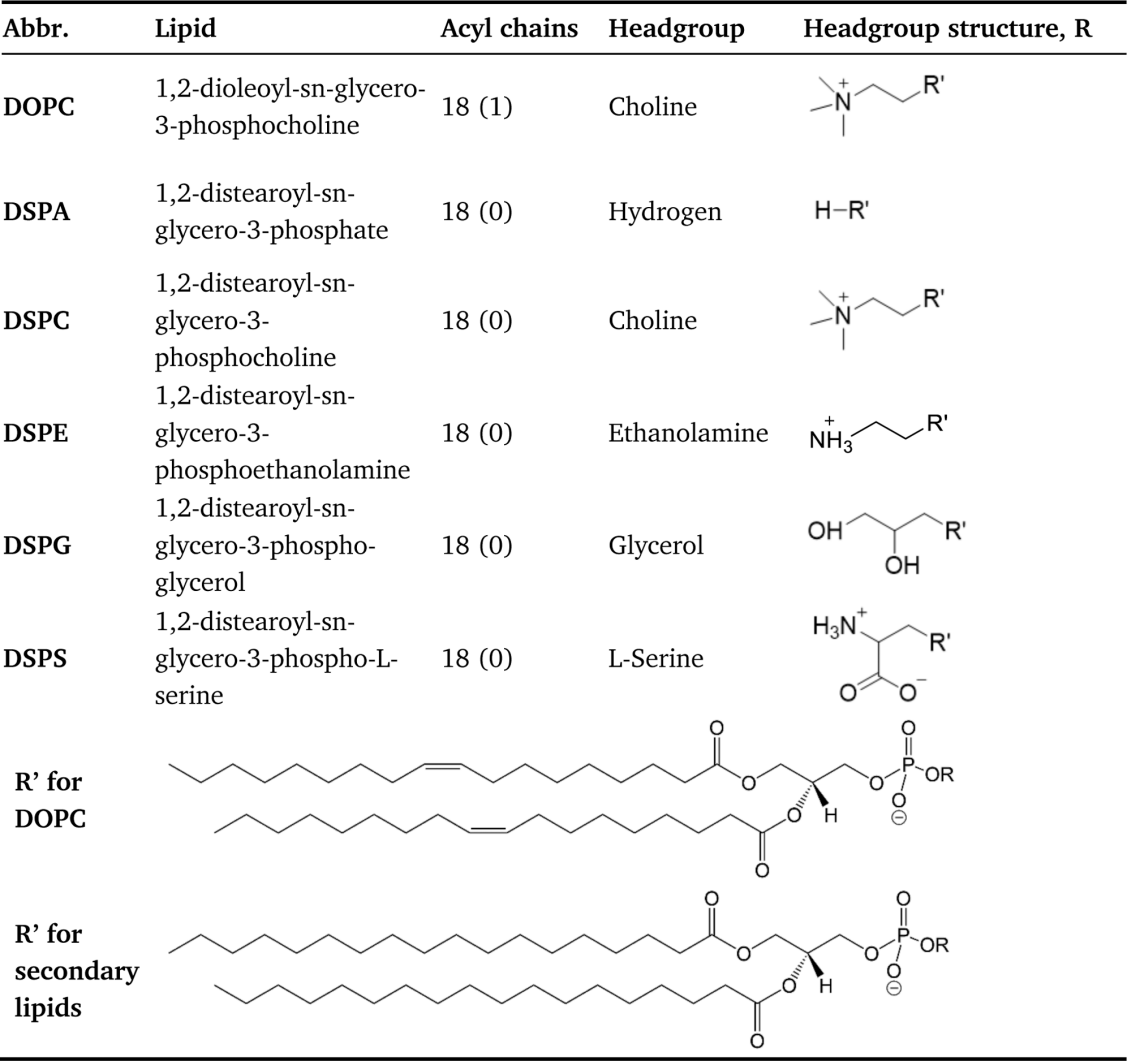
Lipids Investigated in This Work^a^

a Each lipid has two identical acyl chains. The acyl chain column lists the number of carbon atoms and (number of unsaturated bonds) in each acyl chain. As the R′ group has a net charge of −1, in the case of DSPA and DSPG the ‘R’ group includes a Na^+^ ion (not shown) to make the whole lipid electrically neutral.

Results from our previous studies^31,32^ suggest that the mechanical properties of liposomes can be tuned by considering mixtures of lipids with different molecular structures. We previously used molecular dynamics (MD) simulations to study lipid bilayers formed by mixing a long-tailed phospholipid with a single double bond in each of their tails (e.g., DOPC) with a short-tailed saturated lipid with the same headgroup (e.g., 1,2-diheptanoyl-*sn*-glycero-3-phosphocholine, DHPC). Our results^31^ show that systems having 65–75 mol % of DOPC mixed with the shorter lipid DHPC are the least rigid, having area compressibility moduli *K*_*A*_ that are ∼10% smaller than the values observed in pure DOPC bilayers. These results agree with experimental micropipette aspiration measurements^31,32^ showing that the 75:25 liposomes were softer than their pure DOPC counterparts, with stretching moduli that were ∼30% smaller than the values obtained for pure DOPC liposomes. Our results^31^ suggest that mixing DOPC with DHPC alters lipid packing, as the lipid tails are more disordered in the examined mixtures compared to pure DOPC systems. Results from another of our studies^33^ suggest that hydrophobic solute molecules embedded in the lipid tails of liposomes can also alter their stiffness. Antagonist drugs Ki16425 and AM095 can suppress the function of lysophosphatidic acid receptor 1 (LPAR1), which is linked to cancer initiation, progression, and metastasis; however, the hydrophobicity of these drugs limits their in vivo delivery. We have recently reported^33^ the synthesis of two liposomal formulations incorporating AM095 or Ki16425 embedded within a DOPC liposome, as targeted nanocarriers for treatment of breast cancer. Our results indicate that the Ki16425 liposomal formulation exhibited a 50% increase in internalization by cancer cells and a 100% increase in tumor accumulation compared with blank liposome formulations. MD simulations show that the integration of Ki16425 or AM095 makes the liposomes more flexible (58% and 43% smaller *K*_*A*_, respectively) compared to that of the pure DOPC systems.

In this work, we investigated how stiffness in lipid bilayers, where the main component is DOPC, is affected by the presence of other lipids with varying headgroups and saturated acyl chains with the same 18 carbon atoms as DOPC. Using MD simulations with all-atom and coarse-grained models, we determined values for the area compressibility modulus *K*_*A*_, and investigated how interaction energies relate to this variable. We also monitored properties such as order parameters of lipid tails, distances between lipids, and local compositions in our systems. In vitro cellular internalization by normal epithelial and mestastatic breast cancer cells of three different liposome formulations (pure DOPC, DOPC:DSPG, and DOPC:DSPA, the last two with 15 mol % of DSPG or DSPA) are also reported and discussed.

## Materials and Methods

### Computer Simulations

#### Lipids Considered and Details of Model Systems

In this work, we studied bilayers formed by binary mixtures of lipids, where both leaflets have the same composition. All-atom (AA) and coarse-grained (CG) models were used in our simulations. All lipid molecules considered are shown in Table 1, and details of our model systems are presented in Table S1 (Supporting Information). In all our systems, DOPC is termed the primary lipid, as it is the predominant component in our bilayers, whereas the term secondary lipid is used to refer to either DSPA, DSPC, DSPE, DSPG, or DSPS, the minority component in our systems. DOPC has two unsaturated acyl chains, each with 18 carbon atoms and one double bond between the 9th and 10th carbon atoms (Table 1). In analogy to DOPC, the five secondary lipids also have 18 carbon atoms in both their acyl chains, but both tails are saturated (all carbons joined by single bonds) and have different headgroups.

#### All-Atom (AA) MD Simulations

The CHARMM36^34,35^ force field was used to model lipids and ions, and water molecules were represented by the TIP3P model.^36^ To generate the initial structures used in our AA lipid bilayers, we followed the protocols established in our previous work,^31^ using the heterogeneous lipid generation function in Membrane Builder on CHARMM-GUI.^37,38^ A total of 100 lipid molecules were assembled in each leaflet within a rectangular box, with 2.25 nm-thick water layers above and below. The ion concentration was set to 150 mM using sodium chloride to mimic normal serum sodium levels. Bilayers are approximately 8 × 8 nm^2^ in the *x–y* plane. We also ran simulations for pure systems of secondary lipids. In addition, for some systems we ran simulations with larger system sizes, having a total of 144 lipids per leaflet with *x–y* surface areas of ∼10 × 10 nm^2^ (Table S1, Supporting Information).

All all-atom configurations were first relaxed using the 6-step equilibration scheme recommended by CHARMM-GUI, followed by production runs of at least 200 ns. All MD simulations were performed using the NAMD (v3.0-GPU) simulation package^39,40^ with a 2 fs time step. Langevin dynamics and the Nosé–Hoover Langevin Piston algorithm^41,42^ were used to maintain a constant temperature of 298 K and a normal pressure component *P*_*zz*_ of 1 bar, respectively. As the method we chose for the *K*_*A*_ calculation requires area per lipid results from several simulations of a bilayer under several different surface tensions, surface tensions were set to −7, 0, 7, and 15 mN/m for all systems. The surface tension, γ, was determined by calculating the difference between the normal component and the average of the lateral components of the pressure tensor^43−45^
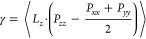
1where *L*_*z*_ is the dimension in the *z*-direction of the simulation box and *P*_*xx*_, *P*_*yy*_, and *P*_*zz*_ are the lateral and normal components of the pressure tensor. In NAMD, we used the *SurfaceTensionTarget* parameter to specify the desired value of the surface tension in our simulated systems. This parameter works in conjunction with the *LangevinPistonTarget* entry to create anisotropic pressure conditions within the simulation box. Specifically, *LangevinPistonTarget* controls the pressure along the *z*-axis *P*_*zz*_ (normal to the membrane plane, which was set at 1 bar for all systems), while *SurfaceTensionTarget* adjusts the values of the lateral pressure components *P*_*xx*_ and *P*_*yy*_ to obtain the desired surface tension value (eq 1). All bonds containing hydrogen atoms were constrained using the ShakeH algorithm.^46^ The particle mesh Ewald (PME) method^47^ with a real-space cutoff at 1.2 nm was used for electrostatic interactions, by applying a shifting function to the electrostatic potential at cutoff distance. van der Waals interactions were cut off at 1.0 nm and smoothly reduced to zero at 1.2 nm using the force-based switching option.

#### Coarse-Grained (CG) MD Simulations

To investigate the possible formation of nanodomains in our systems, we performed CG simulations using the Martini 3.0 model.^48,49^ Our initial structures were assembled using the Martini Maker function of CHARMM-GUI.^37,50,51^ The resulting lipid bilayers had surface areas of approximately 30 × 30 nm^2^ and were hydrated with water and sodium chloride to attain a salt concentration similar to the value considered in our AA simulations. Most of our CG systems consisted of 65% DOPC and 35% of each of the five secondary lipids, but we also ran CG simulations with slightly larger system sizes for corroboration purposes. Table 2 shows CG representations for all lipids, and details of all of our model systems are presented in Table S1 (Supporting Information). CG simulations were carried out using GROMACS^52−54^ (v2018-gpu). The leapfrog algorithm was used for solving Newton’s equation of motion, with an integration time step of 20 fs. Membrane and solvent temperatures were fixed at 298 K individually by the v-rescale thermostat^55^ with a coupling constant of 1 ps. A Berendsen^56^ barostat with a time constant of 5 ps and a Parrinello–Rahman^57^ barostat with a time constant of 12 ps, both with semi-isotropic pressure coupling, were used for equilibration and production runs, respectively. The Lennard-Jones interactions were cut off with a real-space cutoff of 1.1 nm. Electrostatics were considered using the reaction field method^58^ with a real-space cutoff of 1.1 nm and a dielectric constant (ε_*r*_) of 15. Production runs of at least 1 μs were performed for all of our CG systems at a surface tension γ = 0 mN/m.

**Table 2:**
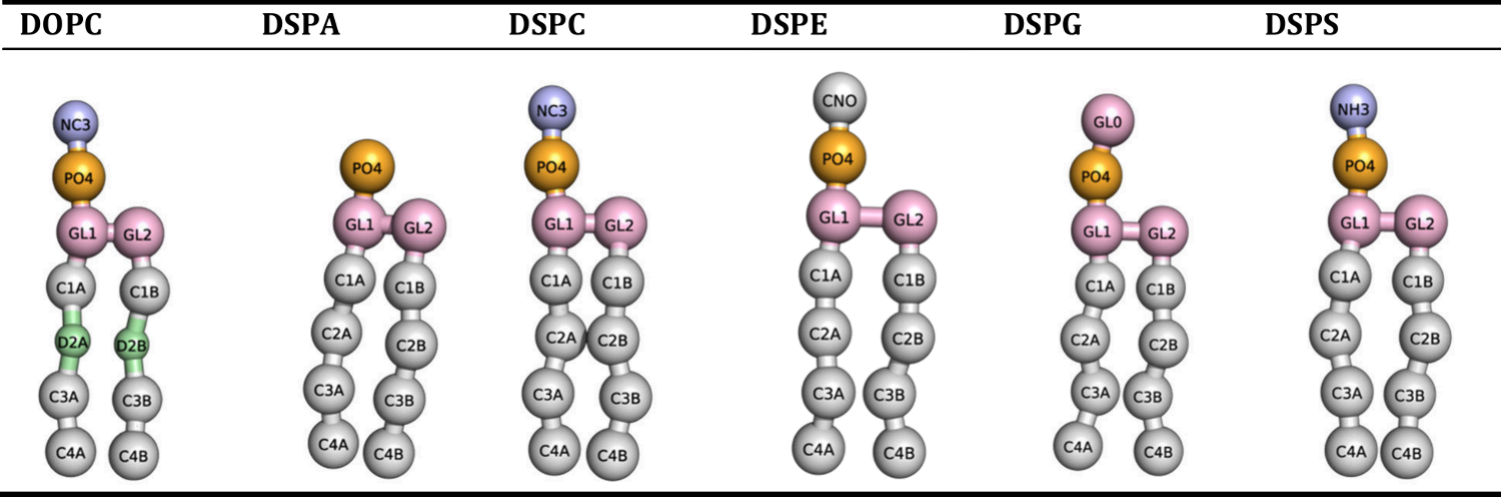
CG Martini Structures of Lipids Used in This Work with Bead Labels

#### Density Functional Theory (DFT) Calculations

We conducted density functional theory (DFT) calculations to study the electrostatic potential (ESP) of the five different lipid headgroups shown in Table 1. Each headgroup structure was attached to a PO_4_^–^ group terminated in a methyl group and protonated according to its protonation state in MD simulations (see Figure 2a). Including the phosphate group in our analysis was motivated by its strong influence on the ESP of the headgroups. For the DFT calculations, the geometric optimization and frequency calculation were performed through the Gaussian16 package,^59^ using the B3LYP^60,61^ functional with a 6-311G** basis set with DFT-D3^62^ corrections. A solvation model based on density (SMD)^63^ was used to implicitly include water molecules. The ESP for five headgroups was calculated by Multiwfn,^64^ and VMD^65^ was used for visual representations.

### Experimental Studies

#### Chemicals

DOPC (1,2-dioleoyl-*sn*-glycero-3-phosphocholine), DSPG (1,2-distearoyl-*sn*-glycero-3-phosphoglycerol), and DSPA (1,2-distearoyl-*sn*-glycero-3-phosphate) were purchased from Avanti Polar Lipids (Alabaster, AL). Ethanol 200 proof, ACS reagent, ≥99.5%, and flow cytometry staining buffer were purchased from Thermo Fisher Scientific (Waltham, MA). DiR (1,1′-dioctadecyl-3,3,3′,3′-tetramethylindotricarbocyanine iodide) was purchased from Biotium (Fremont, CA). Dimethyl sulfoxide (DMSO) was purchased from MilliporeSigma (Burlington, MA).

#### Cell Lines and Cell Culture

Mouse normal epithelial cells (EpH4-Ev), mouse tumorigenic epithelial cells from the mammary gland tissue (4T1), Dulbecco’s Modified Eagle’s medium (DMEM), Roswell Park Memorial Institute (RPMI)-1640 medium, calf bovine serum (CBS), and fetal bovine serum (FBS) were purchased from American Type Cell Culture (Manassas, Virginia). Puromycin was purchased from Thermo Fisher Scientific (Waltham, MA). EpH4-Ev cells were cultured using DMEM medium supplemented with 10% CBS and 1.2 μg/mL puromycin. 4T1 cells were cultured using RPMI-1640 medium supplemented with 10% FBS. Both cell lines were incubated at 37 °C in 95% air, 5% CO_2_, and a humidified atmosphere.

#### Synthesis and Characterization of Liposomes

Three liposomal formulations (L-DOPC, L-DOPC/DSPG, and L-DOPC/DSPA) were synthesized according to the compositions and molar ratios shown in Table 3, using the solvent injection method.^66^ Briefly, DSPG and DSPA were separately dissolved in DMSO at 5 mg/mL each and heated to 65 and 85 °C, respectively. Then a mixture of DOPC and DiR dissolved in ethanol was added to both solutions, and the mixture was stirred for 1 h at the respective temperatures. Afterward, solutions were slowly injected into a phosphate buffered saline (PBS), pH 7.4, heated to the same temperature as the original solution, while constantly stirring. The same method was used to formulate the control at room temperature, without DSPG or DSPA. All formulations were centrifuged in 100 kDa molecular-weight cutoff centrifugal filter tubes at 3000 × *g* for 45 min at 4 °C, to remove the organic solvents, and then resuspended in PBS to yield a total concentration of 2.69 mM.

**Table 3:** Physical Properties of the Liposomal Formulations^a^

**Name**	**Composition**	**Effective Diameter (nm)**	**Polydispersity Index (PDI)**	**Diffusion Coefficient(cm^2^/s)**	ζ-Potential (mV)	**Mobility(μ/s)/(V/cm)**	**Conductance (μS)**
**L-DOPC**	DOPC (99 mol %)	72 ± 2	0.28 ± 0.03	(6.9 ± 0.2) × 10^–8^	2.4 ± 0.1	0.12 ± 0.01	260 ± 4
**L-DOPC/DSPG**	DOPC:DSPG (84:15 mol %)	81 ± 1	0.19 ± 0.01	(6.0 ± 0.1) × 10^–8^	–27 ± 1	–1.43 ± 0.04	262 ± 0
**L-DOPC/DSPA**	DOPC:DSPA (84:15 mol %)	102 ± 3	0.22 ± 0.06	(4.8 ± 0.1) × 10^–8^	–36 ± 1	–1.85 ± 0.06	269 ± 1

a All formulations contained 1 mol % DiR. All values are presented as the mean ± standard deviation. For all measurements, *n* = 3.

The effective diameter, polydispersity, and diffusion coefficient of the formulations (Table 3) were measured by dynamic light scattering (DLS), using a particle size analyzer (Brookhaven Instruments Corporation, Holtsville, NY). Data were collected using BIC particle sizing software. ζ-Potential, mobility, and conductance (Table 3) were measured in deionized H_2_O at 25 °C using BIC phase analysis light scattering (Brookhaven Instrument Corporation).

#### Measurement of the Cellular Internalization of Liposomes

Normal epithelial cells (EpH-4Ev) and metastatic breast cancer (MBC) cells (4T1) were cultured in their respective whole media at 37 °C in 95% air, 5% CO_2_, and humidified atmosphere. Afterward, cells were seeded in 24-well plates at a density of 1 × 10^5^ cells/well. Upon reaching 80% confluency, cells were treated separately, in triplicate, with 0.538 mM in fresh whole media of each of the experimental liposomal solutions and incubated for 4 h under the same conditions as previously mentioned. Next, media were removed and cells detached and resuspended in flow cytometry staining buffer. The mean fluorescent intensity of all samples was measured using a CytoFLEX flow cytometer (Beckman Coulter) to assess cellular uptake.

#### Statistical Analysis

All experimental values were presented as the mean ± standard deviation. Statistical analysis was done using one-way analysis of variance (ANOVA) (α = 0.05, 0.01, 0.001) with post hoc Tukey test. All statistics were run on OriginPro, Version 2023 (OriginLab Corporation, Northampton, MA, USA). Significance was concluded when *P* ≤ α and indicated on the corresponding figures for each experiment as follows: * *p* < 0.05, ** *p* < 0.01, *** *p* < 0.001.

## Results and Discussion

### Area Compressibility Modulus and Electrostatic Potential (ESP) Surfaces of Head and Phosphate Groups

For a lipid bilayer, the area compressibility modulus *K*_*A*_ is a common measure of stiffness, i.e., its ability to resist deformation caused by applied forces. *K*_*A*_ has units of force/distance (e.g., N/m) and determines a membrane’s ability to compress and expand as a compressive or tensile force is applied. Larger *K*_*A*_ values are associated with stiffer lipid bilayers. This property was determined from our AA simulations from the following equations^45,67^
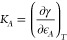
2
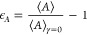
3where γ is the surface tension and ϵ_*A*_ is the area strain. *K*_*A*_ was determined from the slope in a plot of γ vs ϵ_*A*_, by calculating the area per lipid in a lipid bilayer subjected to surface tension values of −7, 0, 7, and 15 mN/m in *NP*_*zz*_*γT* simulations. The average area per lipid was determined by dividing the average *x–y* area of our simulation box by the total number of lipids in one leaflet. Error estimates for *K*_*A*_ were computed from the standard uncertainties in our area per lipid results, as determined from the autocorrelation method described by Grossfield et al.^68^

**Figure 1:**
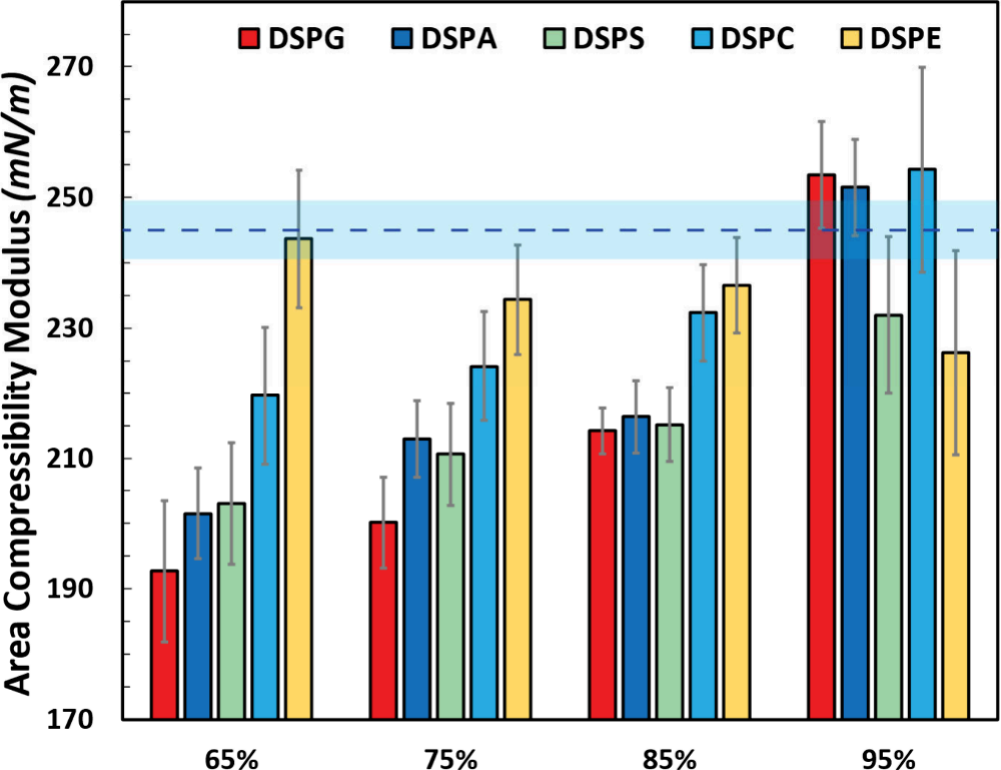
Area compressibility modulus (*K*_*A*_) of lipid bilayers composed of varying DOPC concentrations (65%, 75%, 85%, or 95%) with complementary molar ratios of DSPG, DSPA, DSPS, DSPC, and DSPE. Numerical results are included in Table S2 (Supporting Information). The horizontal line and transparent lake blue shading region indicate the value and uncertainty of *K*_*A*_ of pure DOPC lipid bilayer determined in our previous study.^31^

*K*_*A*_ results for the systems investigated in this work are presented in Figure 1; numerical values are reported in Table S2 (Supporting Information), and plots of the area per lipid of all systems studied are shown in Figure S1. In this figure, values for systems of pure secondary lipids are also presented and are all smaller than the area per lipid obtained for pure DOPC at any given surface tension. In general, all mixtures have an average area per lipid that is smaller than the values observed for pure DOPC at any given value of surface tension (Figure S1). The area per lipid tends to increase as surface tension increases and decreases as the mole fraction of the secondary lipid is raised. In general, the mixtures containing DSPE have the smallest areas per lipid, whereas the DSPG and DSPC mixtures have the largest values at any given composition and surface tension (Figure S1). The area compressibility modulus for a pure DOPC bilayer, *K*_*A*_ = 245.81 ± 9.85 mN/m (Figure 1), was obtained in our previous study^31^ and is in agreement with reported experimental and simulation values.^69−72^ Variations in *K*_*A*_ values are observed upon addition of different mole fractions of secondary lipids, which have different headgroups, and their acyl tails are saturated. We note that the headgroups of DSPC and DOPC are identical with the only difference between these two molecules being that DSPC does not have double bonds in its acyl chains (Table 1). For DOPC:DSPC systems, increasing amounts of DSPC lead to larger reductions in *K*_*A*_, noting also that the area compressibility modulus for the 95:5 DOPC:DSPC system is statistically similar to the pure DOPC value (Figure 1 and Table S2), as confirmed by computation of *p*-values and *t*-scores. Consequently, the reduction in *K*_*A*_ for DOPC:DSPC mixed bilayers is a result of the lack of double bonds in the acyl chains of DSPC as well as composition without any influence from headgroups or length of the carbon tails.

To further explore the effect of headgroups on the area compressibility modulus, we then compared the *K*_*A*_ of any given mixed bilayer with the DOPC:DSPC system of the same molar ratio. For lipid bilayers containing 65% DOPC, the addition of DSPG, DSPA, or DSPS decreases the stiffness further compared to 65:35 DOPC:DSPC, indicating their headgroups (glycerol, hydrogen, and l-serine, respectively; Table 1) have a further positive influence on reducing the stiffness of DOPC-based bilayers. For 65:35 systems, DSPG bilayers show the smallest *K*_*A*_, followed by the DSPA and DSPS systems. The *K*_*A*_ values of the 65:35 DOPC:DSPG, DOPC:DSPA, and DOPC:DSPS systems are 192.7 ± 10.8, 201.5 ± 7.0, and 203.1 ± 3.6 mN/m, respectively (Figure 1 and Table S2). Similar values of area compressibility moduli (i.e., overlapping within the error bars) were obtained when we ran simulations of these systems with larger sizes (144 vs 100 lipids per leaflet; Tables S1 and S2). These values are smaller than the *K*_*A*_ obtained for the 65:35 DOPC:DHPC bilayer, *K*_*A*_ = 220.5 ± 3.8 mN/m, which was the system that had the smallest area compressibility modulus in our previous study,^31^ where we mixed DOPC with saturated lipids with shorter acyl tails (seven carbon atoms for DHPC) and the same choline headgroup as DOPC. In contrast to all the systems mentioned above, the 65:35 DOPC:DSPE mixture and the pure DOPC system have statistically similar values of *K*_*A*_, which are both larger than the *K*_*A*_ of the DOPC:DSPC mixture. This observation suggests that the headgroup of DSPE (ethanolamine) counteracts the stiffness-reduction effect of its saturated acyl chains, thus resulting in a minimal overall influence on the *K*_*A*_ value compared with a pure DOPC bilayer. This observation is interesting and surprising as DSPE and its unsaturated counterpart DOPE (1,2-dioleoyl-*sn*-glycero-3-phosphoethanolamine) have membrane fusogenic characteristics.^73^

For systems of lipid bilayers composed of 75% and 85% DOPC, our observations show that the *K*_*A*_ trend among the five systems is similar to that observed in the 65% systems (i.e., DSPG < DSPA < DSPS < DSPC < DSPE, Figure 1). We note that the *K*_*A*_ values for the DSPA and DSPS systems are very similar for any of these three given compositions. For the 85:15 systems, the DSPG, DSPA, and DSPS bilayers have the same value of area compressibility modulus within the reported uncertainties (Figure 1). Furthermore, the *K*_*A*_ values for the 75:25 and 85:15 DOPC:DSPG, DOPC:DSPA, and DOPC:DSPS systems are smaller than the area compressibility moduli of DOPC:DSPC at the same compositions. This observation suggests that these particular headgroups consistently influence the stiffness of the DOPC bilayers at these compositions. For all bilayers containing 95% DOPC, the values of *K*_*A*_ are statistically similar to the area compressibility modulus of a pure DOPC bilayer, as confirmed by computation of *p*-values and *t*-scores (Table S2). The molar ratio of secondary lipids also impacts the stiffness of the bilayers. Decreasing *K*_*A*_ values are observed as the molar ratio of DSPG, DSPA, DSPC, and DSPS increases (Figure 1), which suggests that further reductions in *K*_*A*_ could be achieved by increasing the mole fraction of the secondary lipids beyond 35%, potentially to the detriment of the stability of the liposomes in experiments. For DOPC:DSPE systems, the results shown in Figure 1 and Table S2 in general suggest that varying the amount of DSPE does not lead to significant changes in the values of *K*_*A*_, which tend to be statistically similar to the area compressibility modulus observed for pure DOPC bilayers.

**Figure 2:**
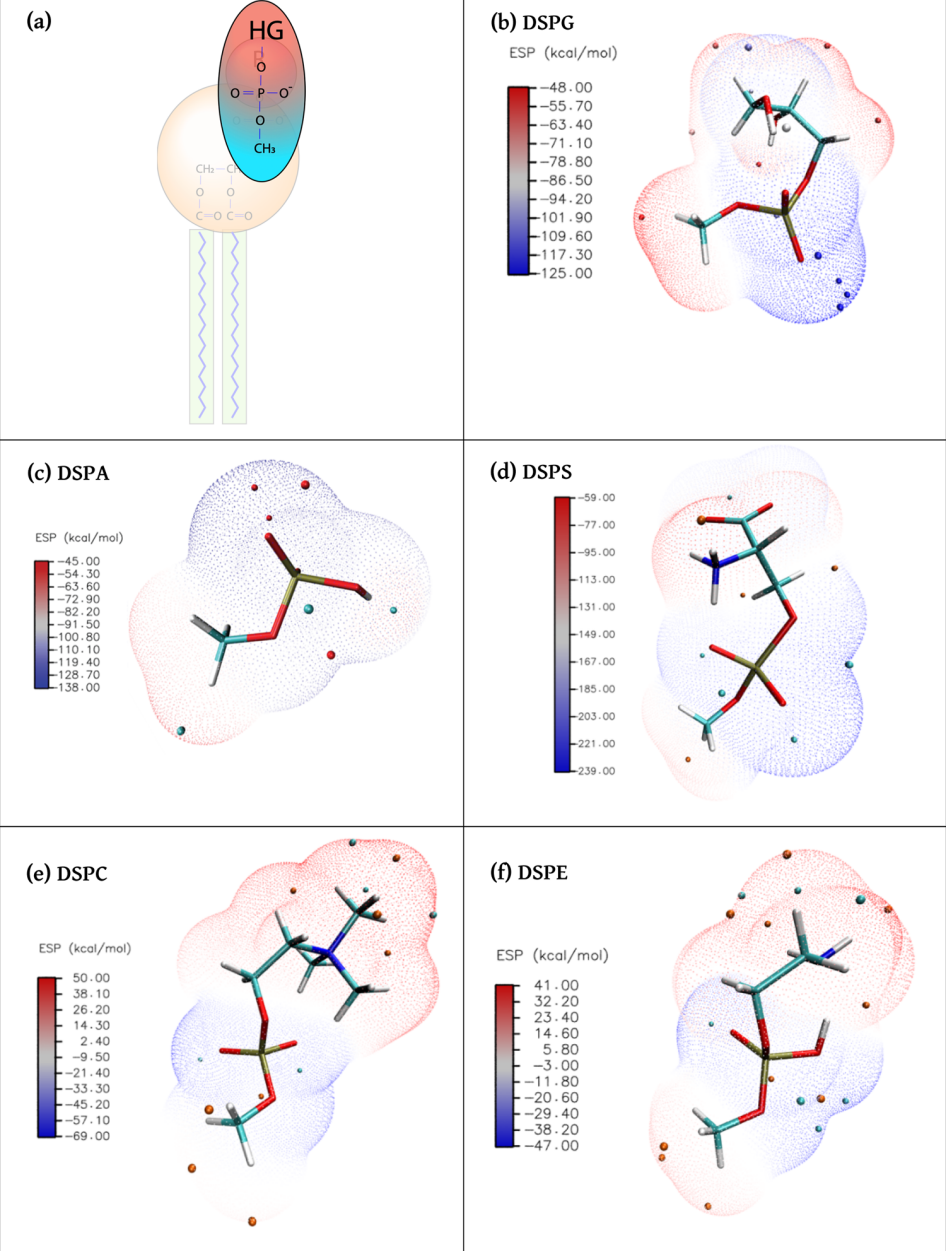
Electrostatic potential (ESP) surfaces. (a) Each headgroup structure was attached to a phosphate PO_4_^–^ group terminated in a methyl group. (b–f) ESP for the five different lipid head and phosphate groups studied; orange and cyan dots depict the locations of local maxima and minima at the ESP surface, respectively. Note that for DSPG, DSPA, and DSPS the ESP values are all negative, but for DSPC and DSPE they vary from negative to positive values.

We performed DFT calculations to investigate the electrostatic potential (ESP) surfaces of the five different lipid headgroups studied (Table 1). Each headgroup structure was attached to a phosphate (PO_4_) group terminated in a methyl group (Figure 2a), as the PO_4_ group is expected to have a strong influence on the headgroup ESP. These results are presented in Figure 2b–f. The ESP surfaces provide information about the distribution of charges in the head and phosphate groups studied, hinting also at how they would interact with nearby polar molecules. We first note that the ESP for glycerol (DSPG), hydrogen (DSPA), and l-serine (DSPS) range entirely between negative values (see color bars in Figure 2b–d), whereas the ESP for both choline (DSPC) and ethanolamine (DSPE) span from negative to positive values. Looking at the results shown in Figure 1, mixing DOPC with DSPG, DSPA, and DSPS leads to the largest reductions in *K*_*A*_, whereas the smallest changes in area compressibility moduli are observed in mixtures of DOPC with either DSPC or DSPE. These observations suggest that the variations in *K*_*A*_ values might be linked to the energetic interactions between the different lipids, which we analyze and discuss in the next section.

### Interaction Energies

**Figure 3:**
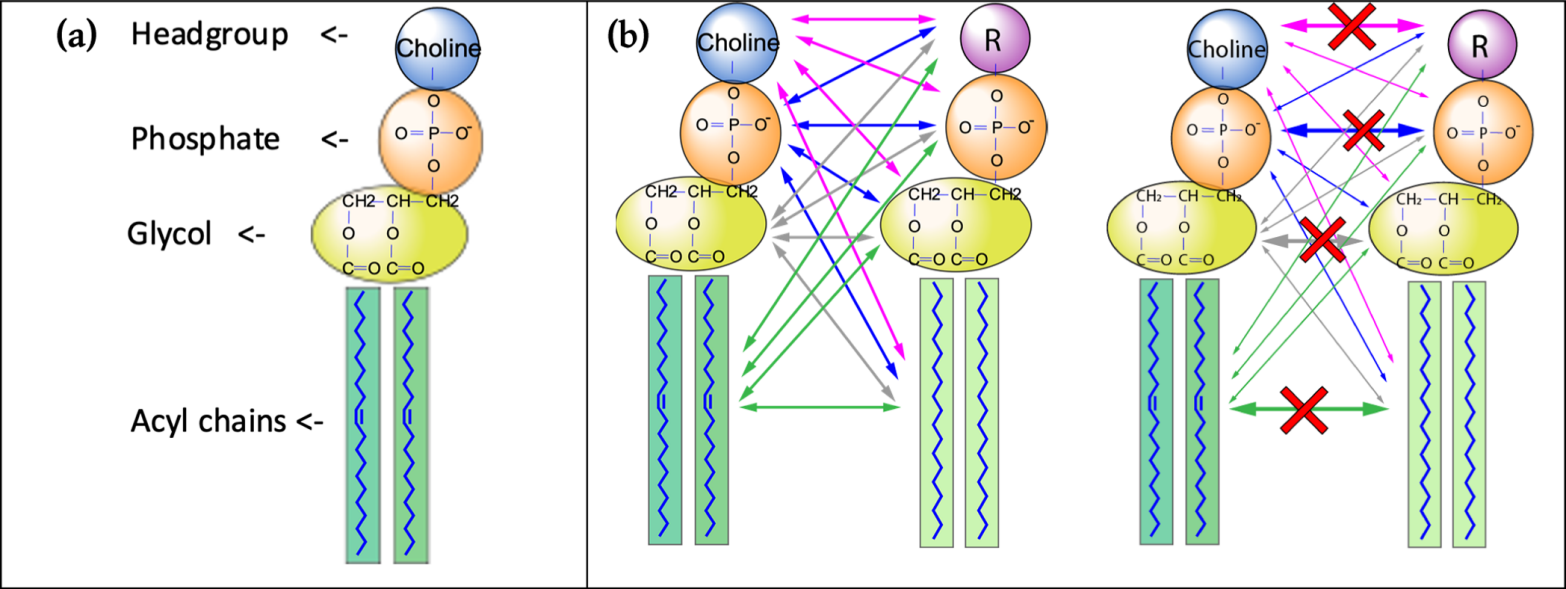
(a) Different subunits in a DOPC lipid molecule: headgroup, phosphate group, glycol group, and acyl chains. (b) Total interaction energies (left) and partial interaction energies (right). Head–head, phosphate–phosphate, glycerol–glycerol, and tail–tail interactions between different lipid molecules are not added when computing partial interaction energies.

**Figure 4:**
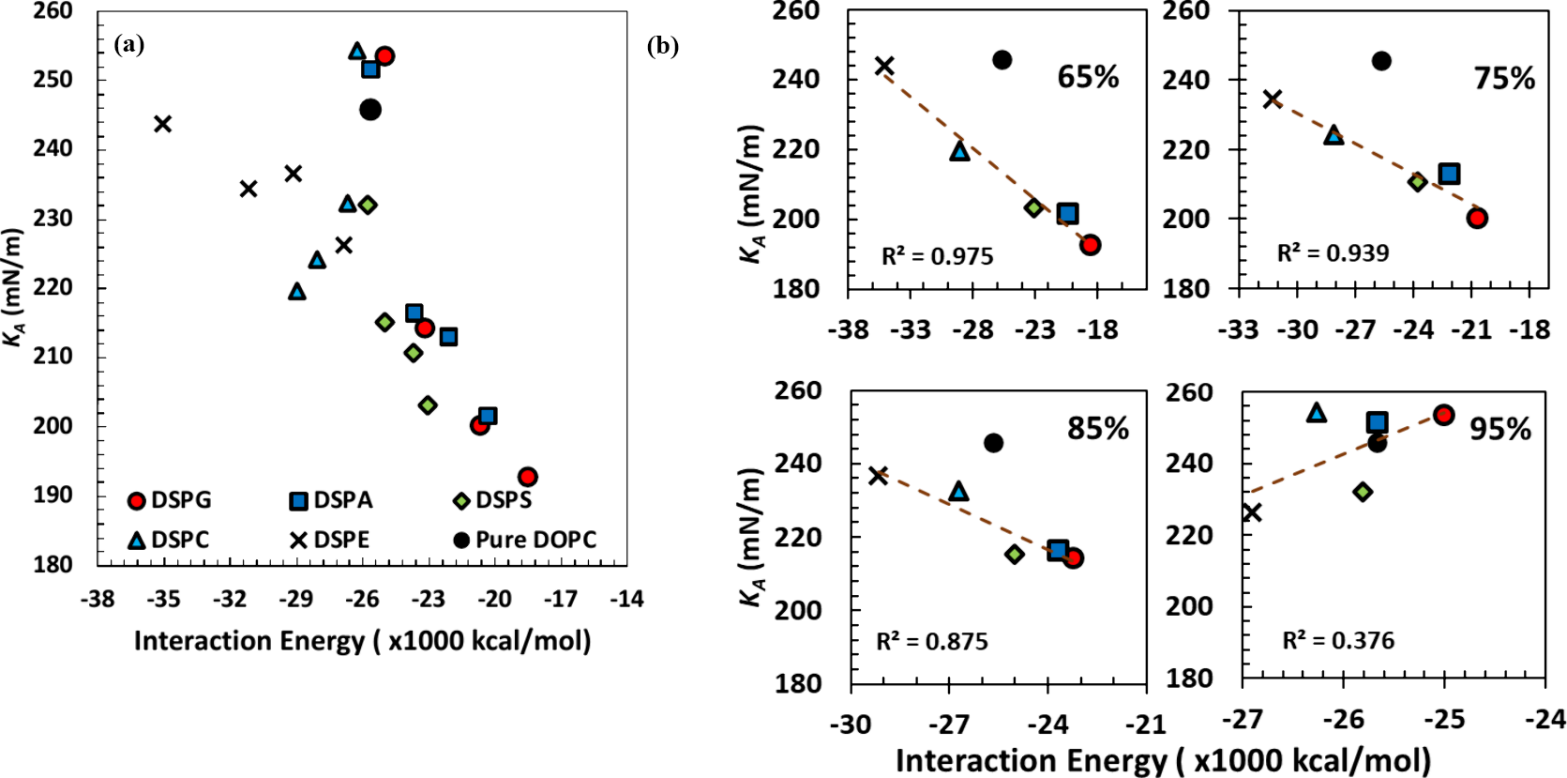
*K*_*A*_ vs partial (*i*–*j*) interaction energies between lipids. (a) All systems and (b) 65%, 75%, 85%, and 95% DOPC systems shown separately.

**Figure 5:**
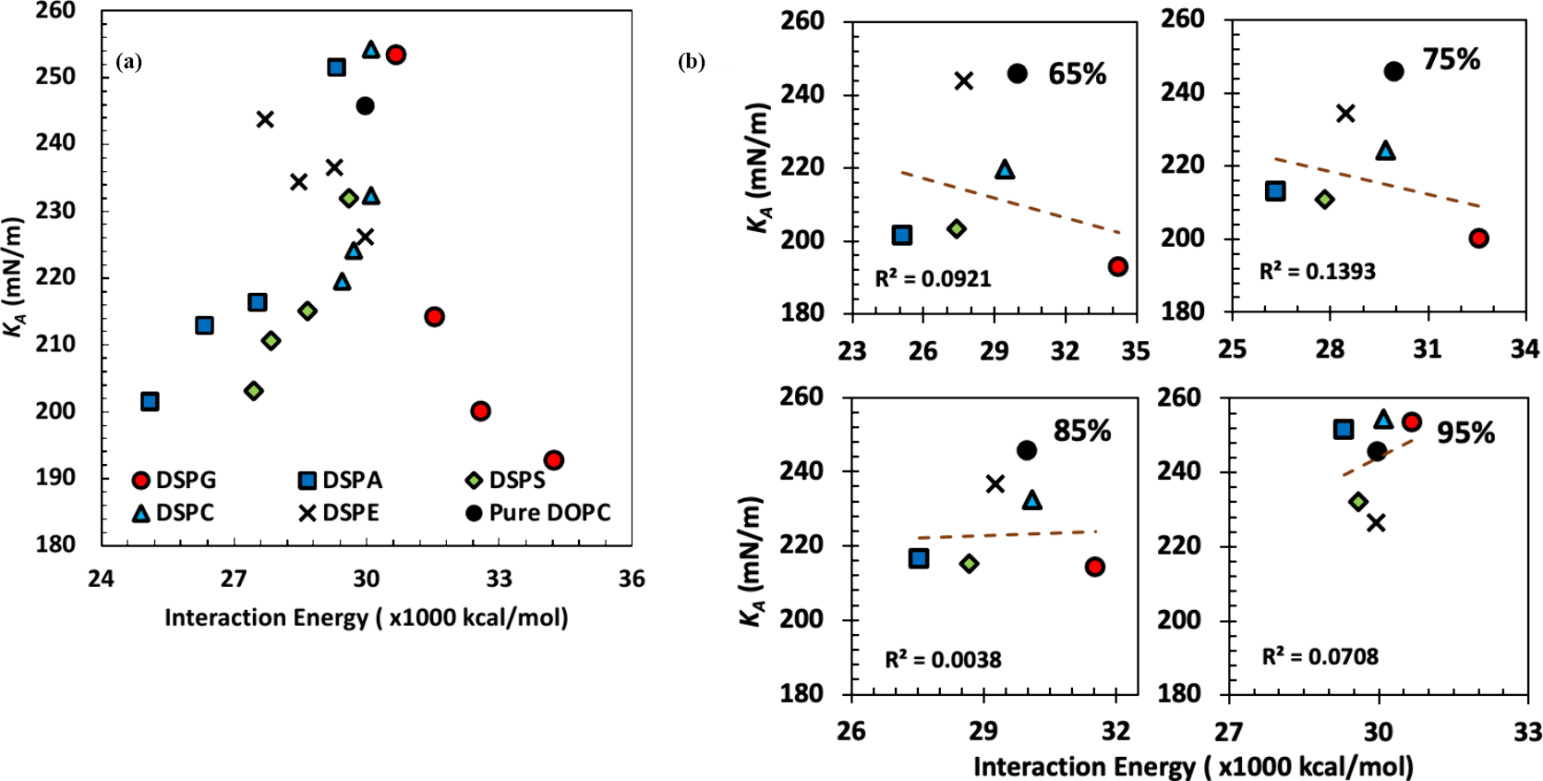
*K*_*A*_ vs total interaction energies between lipids. (a) All systems and (b) 65%, 75%, 85%, and 95% DOPC systems shown separately.

**Figure 6:**
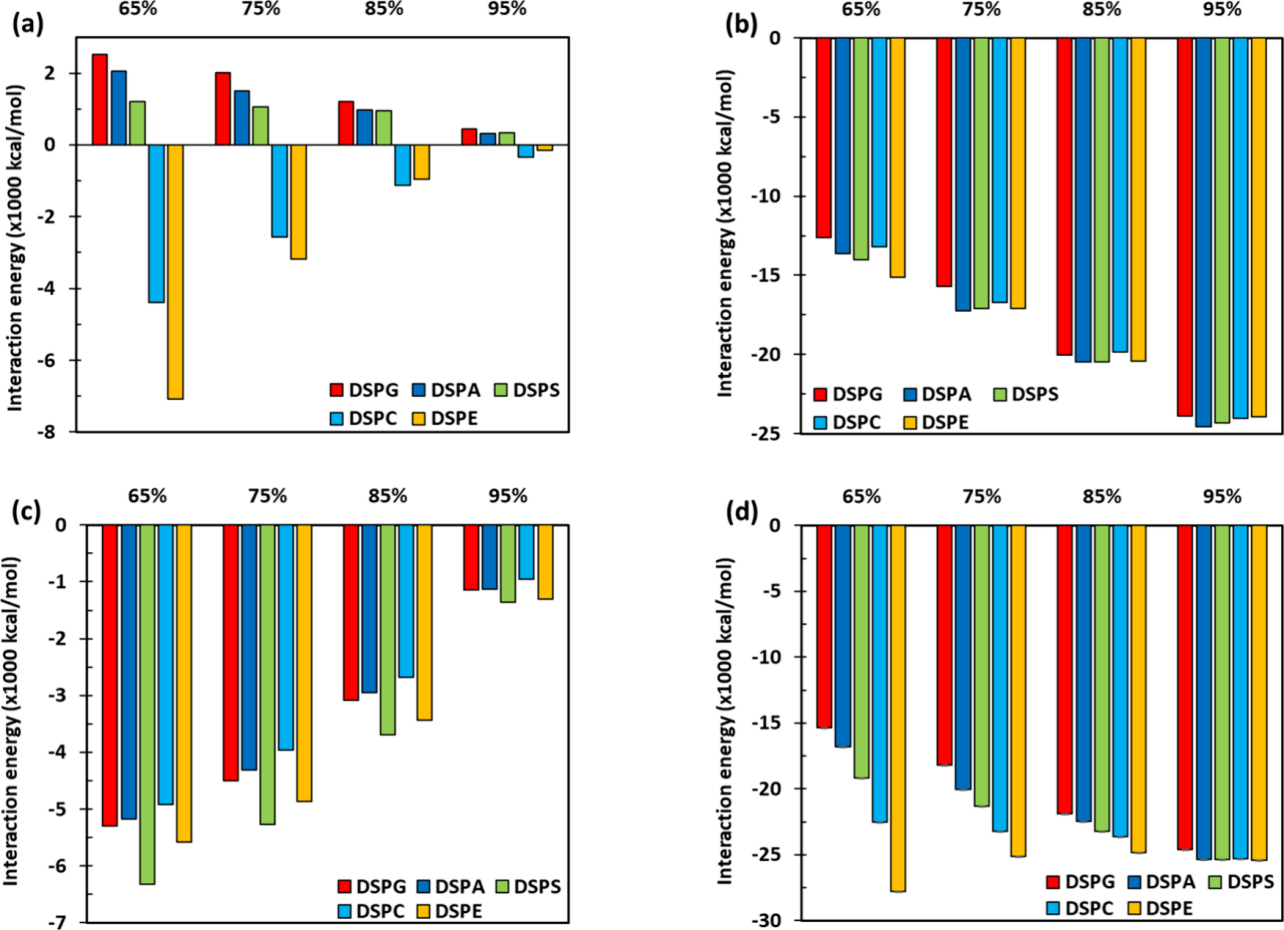
Decomposed partial interaction energies in mixed lipid bilayers at a surface tension of γ = 0 mN/m. The plots show the pairwise partial interaction energies between (a) secondary lipids with secondary lipids, (b) DOPC–DOPC, (c) DOPC with secondary lipids, and (d) overall lipid–lipid. Numerical results are included in Table S3 (Supporting Information).

**Figure 7:**
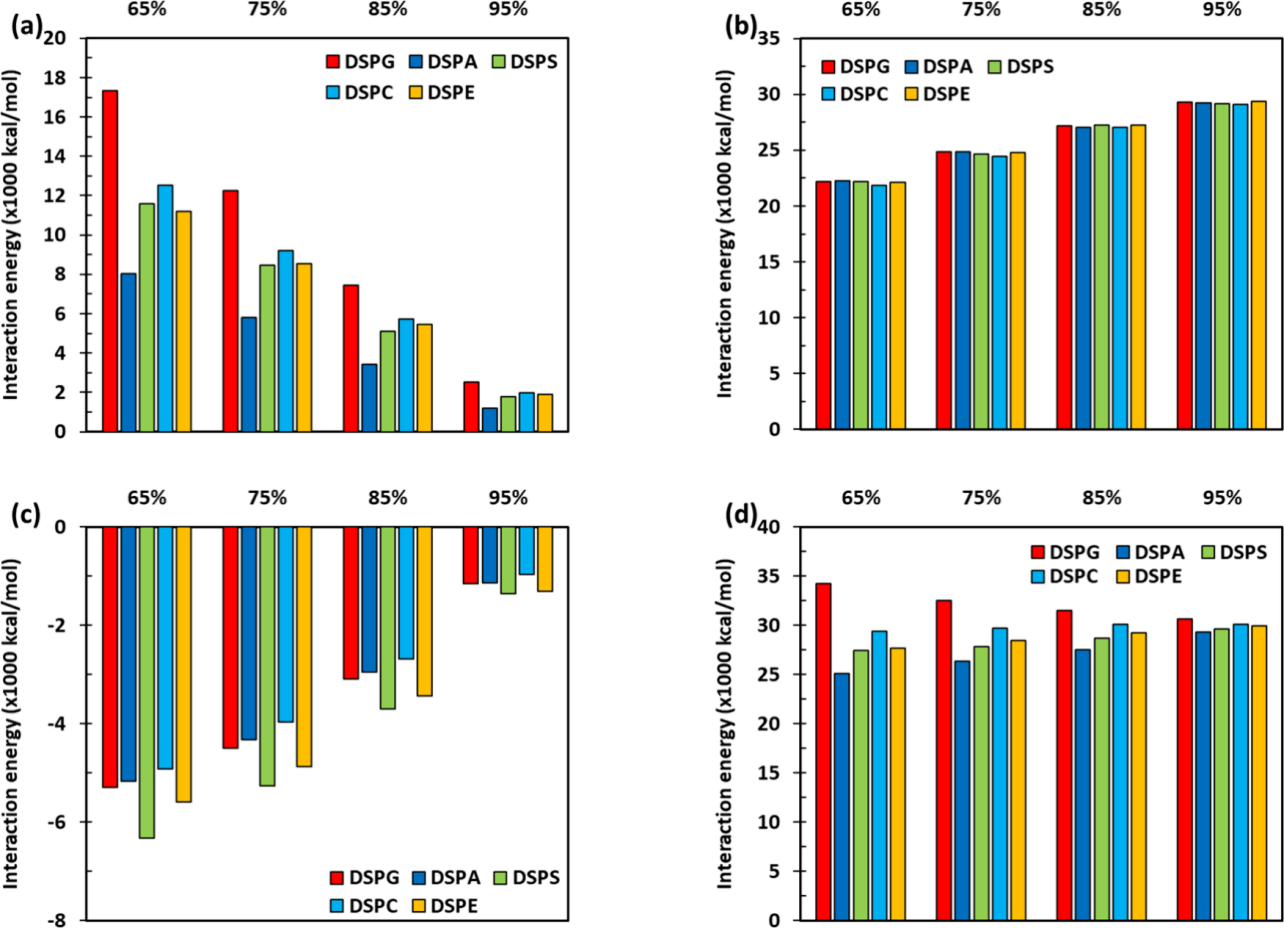
Decomposed total interaction energies in mixed lipid bilayers at a surface tension of γ = 0 mN/m. The plots show the pairwise total interaction energies between (a) secondary lipids with secondary lipids, (b) DOPC–DOPC, (c) DOPC with secondary lipids, and (d) overall lipid–lipid. Numerical results are included in Table S4 (Supporting Information).

We computed the interaction energies between lipids on a group level, as we divided a lipid molecule into four distinct subunits: headgroup, phosphate group, glycol group, and acyl chain (Figure 3a). Subsequently, we assess the interaction energies occurring among these individual groups. We calculated two types of energies between pairs of lipids, namely, total and partial (*i*–*j*) interaction energies (Figure 3b). Total interaction energies sum over all interactions between the 4 groups of all lipid molecules, while partial (*i*–*j*) interaction energies only include interactions between two different groups *i* and *j*, excluding the *i*–*i* and *j*–*j* interactions from the sum. For example, interactions between two headgroups belonging to any two lipid molecules of the same or different species are not included, but interactions between headgroups and acyl chains of any two lipid molecules are included in the computation of partial interaction energies (Figure 3b). Phosphate–phosphate interactions involve Coulomb interactions between two groups that are negatively charged; likewise, most headgroup–headgroup interactions involve two groups that are positively charged. These Coulomb interactions will, therefore, result in positive energy values (repulsion). Therefore, values for partial energies in Figures 4 and 6 are mostly negative, whereas values for the total energies in Figures 5 and 7 are positive.

Figures 4 and 5 show *K*_*A*_ against the partial and total interaction energies, respectively. Figure 4a illustrates how the partial interaction energies among lipid molecules relate to *K*_*A*_ for all compositions examined, whereas results for different systems at the same mole % of DOPC are shown in Figure 4b; all energy data is presented in Table S3 (Supporting Information). Similar results are shown in Figure 5a and b and Table S4 but for the total interaction energies. Results for pure DOPC are included in both figures. In Figure 4a, *K*_*A*_ and partial interaction energy for most systems seem to vary linearly with a negative slope, i.e., with *K*_*A*_ becoming smaller as the partial interaction energy increases (becomes less negative). In contrast, no linear trends are observed between *K*_*A*_ and the total interaction energies for all systems in the results presented in Figure 5a; however, in this figure, linear trends are apparent within systems containing the same secondary lipid at different molar ratios. Coming back to partial energies, in Figure 4b we observe again a linear relationship with a negative slope between *K*_*A*_ and partial energies for each mole % of DOPC, with the exception of the 95% DOPC systems, in which a positive slope is observed. In all cases, the largest interactions (most negative partial energies) are observed for DSPE systems, followed by DSPC, DSPS, DSPA, and DSPG (Figure 4a,b). The linear relationship between partial interaction energies and *K*_*A*_ seems to be more pronounced with increasing molar ratios of secondary lipids, as determined by the *R*^2^ values presented in each subfigure, varying from *R*^2^ = 0.975 in the 65% DOPC systems, to a very weak linear relationship (*R*^2^ = 0.376) in the 95% DOPC systems (Figure 4b). These observations could be beneficial for trying to predict target properties using segmented interaction energies by training and using a machine learning model. In contrast, no linear trends are observed between *K*_*A*_ and total interaction energies in the results shown in Figure 5b, where all *R*^2^ values are close to zero at each mole % DOPC considered.

Why do some of the interaction energies show a linear relationship with *K*_*A*_? An increase in interaction energies between lipids (i.e., making energies more negative) can lead to a reduction in the mobility or flexibility of the system, resulting in an increase in its stiffness. For example, raising the cross-linking density in a polymer network may produce an increase in interaction energies, which can lead to a reduction in chain mobility and an increase in stiffness.^74^ The lipid–lipid partial and total interaction energies can be decomposed into contributions DOPC–DOPC, DOPC–secondary lipids, and secondary lipids–secondary lipids. This decomposition is done to delve deeper into the interactions among lipids and attempt to explain the results shown in Figures 1, 4, and 5, as the importance of these contributions will vary as the molar ratio of secondary lipids increases in our examined mixtures. The decomposed partial interaction energies (which exclude interactions between same groups, Figure 3b) are presented in Figure 6, while the decomposed total interaction energies are shown in Figure 7; all data are shown in Tables S3 and S4, respectively (Supporting Information). The results presented in Figure 6a show important variations in the secondary lipid–secondary lipid partial energies among the different systems. In the 65:35 systems, the DSPG–DSPG partial energies in their mixture with DOPC are the most positive, signaling the weakest attractive partial interactions among secondary lipids, whereas the DSPE–DSPE partial energy in the DOPC:DSPE system is the most negative, indicating the strongest attractive partial interactions between secondary lipids. The difference in partial interaction energies between secondary lipids in these two systems is approximately 9.5 × 10^3^ kcal/mol, which is comparable to the magnitude of the DOPC–DOPC partial interactions in all 65:35 systems (Figure 6b) and is larger than the DOPC–secondary lipid partial interactions shown in Figure 6c for mixtures with 65 mol % DOPC. The partial interactions between secondary lipids (Figure 6a) approach zero as the mole fraction of DOPC increases. The partial interactions among secondary lipids can be split into two groups, one in which these energies are positive (DSPG, DSPA, DSPS) and another in which those interactions have negative values (DSPC, DSPE, Figure 6a). Before, we commented that the ESP values for DSPG, DSPA, and DSPS were all negative (Figure 2), whereas for DSPC and DSPE the ESP values ranged between negative and positive values; these observations suggest analogies between the results shown in Figures 2 and 6a. Now looking at the DOPC–DOPC and DOPC–secondary lipid components of the partial interaction energies (Figure 6b,c), the values observed are relatively similar among systems with the same mole fraction of DOPC, indicating that these contributions are not significantly different among the secondary lipids considered. The DOPC–DOPC partial interactions become more negative, and the DOPC–secondary lipid partial energies have smaller absolute values as the mole fraction of the DOPC increases. For the overall lipid–lipid partial interactions shown in Figure 6d, which result from adding the values presented in Figure 6a–c, we note that these partial energies increase (become more negative) as the molar ratio of DOPC increases for all mixtures, except for the DOPC:DSPE systems. The 65:35 DOPC:DSPE system has the strongest lipid–lipid partial interactions, but these interactions vary slightly as the amount of DOPC increases; we also note that, for the DOPC:DSPE system, the *K*_*A*_ results were statistically similar upon variations in mole fraction (Figure 1). For the other mixtures, in contrast, *K*_*A*_ increases (Figure 1) and the lipid–lipid partial energies become stronger (Figure 6d) as the mole fraction of DOPC is raised.

Results for the decomposition of the total interaction energies are shown in Figure 7. For a given lipid mixture, clear trends are observed as the mole fraction of DOPC increases: the total interactions between secondary lipids become stronger (i.e., less positive, Figure 7a) and the total energies between DOPC and secondary lipids become weaker (i.e., less negative, Figure 7c), whereas the DOPC–DOPC total energies become more positive (Figure 7b). However, the overall lipid–lipid total energy (Figure 7d) slightly decreases with increasing DOPC mole fraction for the DSPG systems, but it slightly increases for the rest of the systems. Linking to the area compressibility modulus results shown in Figure 1, we do not observe clear relationships between *K*_*A*_ with the total interaction energies shown in Figure 7. In contrast, trends are observed between the *K*_*A*_ (Figures 1 and 4) and partial interaction energies (Figure 6).

**Figure 8:**
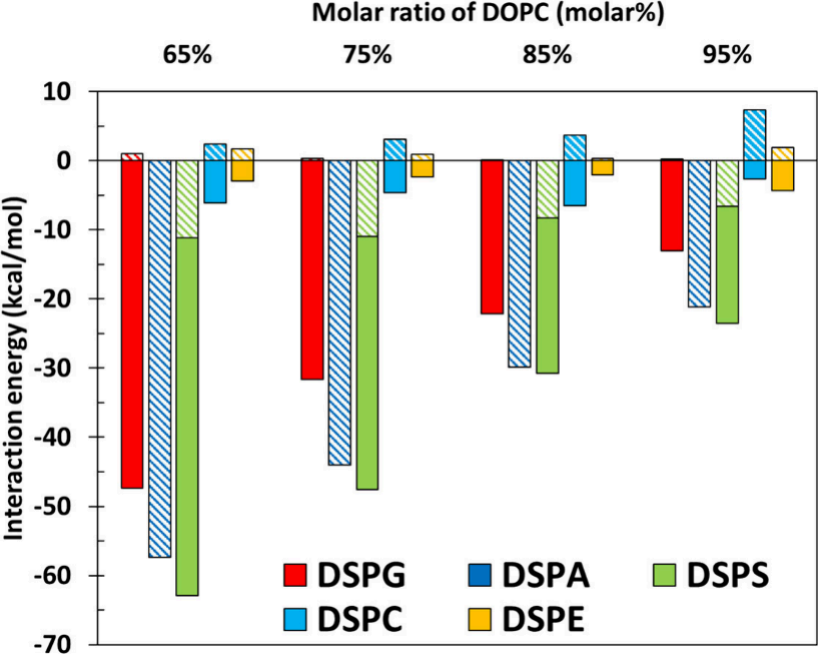
Interaction energy between salt (sodium chloride) ions and head/phosphate groups of secondary lipids in the five DOPC mixtures studied. Solid bars represent the interaction energies between salt ions and the lipid headgroups, while bars with diagonal stripes indicate the interaction energies between salt ions and phosphate groups. For DSPA, the headgroup is a lone hydrogen atom (Table 1), so results for these systems do not include a solid bar.

Sodium chloride ions engage predominantly with the head and phosphate groups of lipids and have been found in previous studies to influence the structure of mixed lipid bilayers.^75^ As mixing DOPC with lipids with different headgroups produces changes in *K*_*A*_ (Figure 1), it is interesting to monitor the interactions between NaCl and the lipid head and phosphate groups. Results for these salt–secondary lipid interactions are presented in Figure 8, where solid and striped bars represent the energies between ions and the headgroups and phosphate groups of the secondary lipids. As DSPA “lacks” a headgroup (it is only a hydrogen atom, Table 1), in Figure 8 we only show the ion–phosphate interactions. Numerical results are shown in Table S5 (Supporting Information). In analogy to the results shown in Figures 2 (ESP) and 6a (partial energies between secondary lipids), we observe two distinct groups of systems based on their interactions with salt ions, one where the energies are large and negative (DSPG, DSPA, and DSPS) and another group with energies that are smaller in magnitude (DSPC and DSPE). The ion–headgroup pair is the major contributor to the interactions between ions and lipids in DSPG and DSPS systems, whereas the ion–phosphate interactions are much larger in the DSPA system compared to the other mixtures. Focusing now on the 65% DOPC systems, we observe that the interaction strength between ions and the head and phosphate groups of lipids can be ordered as DSPS > DSPA > DSPG > DSPC > DSPE, which aligns with the order of the most negative values in the color bars of the ESP surfaces shown in Figure 2. Furthermore, for DSPG, DSPA, and DSPS systems, the energies shown in Figure 8 become smaller in magnitude as the mole fraction of DOPC increases. In contrast, for DSPC and DSPE systems these energies fluctuate slightly as the DOPC content is raised.

Several studies investigated how buffers or ion concentrations affect the bending rigidity of lipid bilayers using experiments and simulations.^76−78^ Notably, Lu et al. discovered that the bending rigidity of anionic DPPG bilayers decreases by approximately six times upon increasing the NaCl concentration.^77^ These findings align with our results, in which lipid bilayers having strong interactions with ions (Figure 8) have smaller values of *K*_*A*_ (Figure 1). The increased softness of these bilayers suggests that ion–lipid interactions play a significant role in modulating the mechanical behavior of lipid membranes. These observations also suggest that the ion concentration can alter the mechanical properties of lipid bilayers. Further investigation can provide insights into the underlying mechanisms governing the mechanical properties of lipid membranes and add to our understanding of biophysical processes involving lipids and ions.

### Order Parameters

Properties of lipid nanoparticles and bilayers can be interpreted by investigating the conformation of lipid acyl chains.^79−82^ In this work we studied the carbon–carbon order parameter *S*_*C*_, defined as
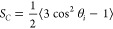
4where θ_*i*_ is the angle between the membrane normal (i.e., the *z*-axis of the simulation box) and the vector joining carbon atoms *C*_*i*–1_ to *C*_*i*+1_ of the lipid tail.

**Figure 9:**
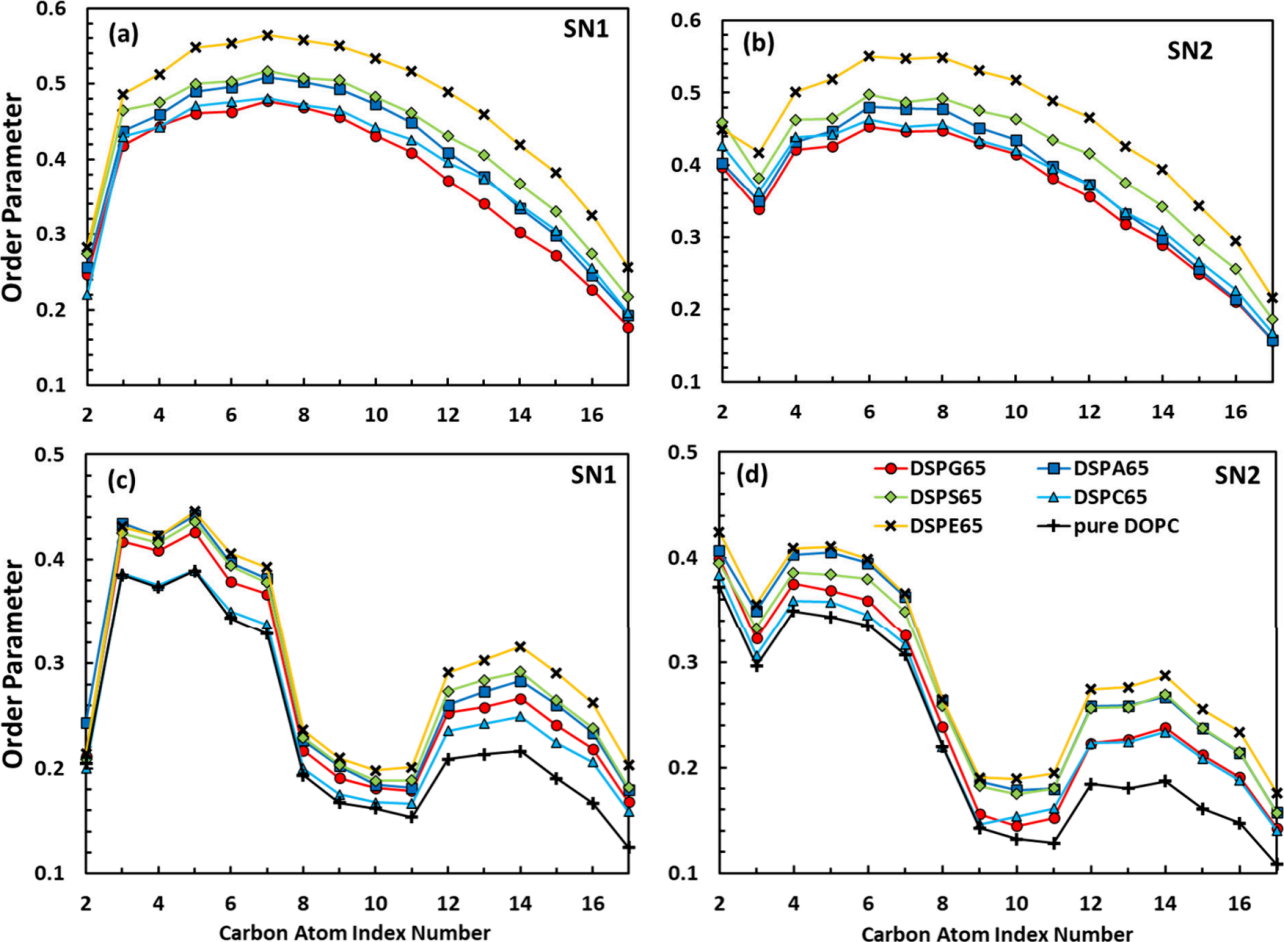
Order parameter *S*_*C*_ for the two lipid tails SN1 and SN2 in our 65:35 lipid bilayer mixtures at a surface tension of γ = 0 mN/m, for the secondary lipids (a, b) and for DOPC (c, d); results for a pure DOPC bilayer are also included in (c) and (d). All figures share the legend shown in (d).

In our previous work,^31^ we found that adding secondary lipids with short, saturated acyl chains to DOPC bilayers led in general to reductions in the order parameter *S*_*C*_ of all lipids. This observation indicates that adding secondary lipids with short tails to DOPC bilayers disrupts lipid packing and increases disorder in the longer, unsaturated acyl chains of DOPC, which was associated with decreased stiffness in the mixed lipid bilayers. These effects should not be present in the systems studied in this work as DOPC and all secondary lipids here have 18 carbon atoms in their acyl tails. Therefore, any changes in the spatial layouts of the tails would be due to the different headgroups in the secondary lipids and to the presence of a carbon–carbon double bond in both DOPC tails. The *S*_*C*_ for both tails SN1 and SN2 of the secondary lipids in 65% DOPC bilayers are shown in Figure 9a,b, while equivalent results for DOPC in the same mixed lipid bilayers are presented in Figure 9c,d; results for a pure DOPC bilayer are also included in Figure 9c,d. Equivalent results for the rest of the mixtures studied are shown in Figures S2–S4. From Figure 9a,b, the smaller values of *S*_*C*_ in the tails of the secondary lipids are observed for DSPG and DSPC, followed by DSPA, DSPS, and DSPE. These trends align in general with the transition temperatures between the gel and liquid crystalline phases for the pure secondary lipids, where DSPG and DSPC have the lowest transition temperatures (55 and 56 °C) compared to DSPA, DSPS, and DSPE (75, 68, and 74 °C, respectively). When trying to find relations between the *S*_*C*_ values for the secondary lipids (Figure 9a,b) and the *K*_*A*_ results shown in Figure 1, we note that the *S*_*C*_ of DSPG was the smallest (more disordered acyl tails) and that of DSPE was the largest (tails tend to align more vertically); likewise, among the 65:35 mixtures, the smallest and largest *K*_*A*_ values were found in the DSPG and DSPE systems (Figure 1). However, for the other systems (DSPC, DSPA, and DSPS), no apparent trends are observed between the order of *S*_*C*_ (DSPC < DSPA < DSPS, Figure 9a,b) and *K*_*A*_ (DSPA < DSPS < DSPC, Figure 1).

The results shown in Figure 9c,d indicate that the addition of lipids with different headgroups but the same length of lipid tails as DOPC (but no double bonds) leads to an increase in the *S*_*C*_ of both DOPC acyl chains, as compared to the results obtained in a pure DOPC bilayer. This observation contrasts what was found in our previous study,^31^ where adding lipids with the same headgroup as DOPC but with shorter saturated tails led to an increased disorder (smaller *S*_*C*_) in the DOPC lipid tails, which was linked to a reduction in area compressibility modulus *K*_*A*_ in mixed systems. In contrast, here no apparent relation is found between the trends for the order parameters of DOPC shown in Figure 9c,d (i.e., pure DOPC < DSPC < DSPG < DSPA < DSPS < DSPE) and the *K*_*A*_ trends from Figure 1 (DSPG < DSPA < DSPS < DSPC < DSPE < pure DOPC). It is interesting to compare the *S*_*C*_ results obtained for pure DOPC with the equivalent results for the primary lipid in the 65:35 DOPC:DSPC system (Figure 9c,d), as these lipids only differ by the presence of a double bond linking the 9th and 10th carbons in each of the DOPC tails. From Figure 9c,d starting from the second carbon atom, *S*_*C*_ in both systems overlap until we approach the ninth carbon atom, where *S*_*C*_ in the DOPC:DSPC system becomes increasingly larger compared to the values found in pure DOPC. For mixtures at the other compositions examined, the results shown in Figures S2c,d, S3c,d, and S4c,d for DOPC follow similar trends as the data presented in Figure 9c,d, but in general the increase in *S*_*C*_ for DOPC tends to diminish as the mole fraction of secondary lipid decreases. For the secondary lipids, in general, the values of *S*_*C*_ tend to become smaller as the mole fraction of secondary lipids decreases. No other patterns can be established for the mixtures examined; DSPG continues to have the lowest values of *S*_*C*_ in the 75:25 mixtures (Figure S2a,b), but the order parameter values tend to be similar among all secondary lipids in 85:15 and 95:5 mixtures (Figures S3a,b and S4a,b).

### Lipid Spacing Distribution

**Figure 10:**
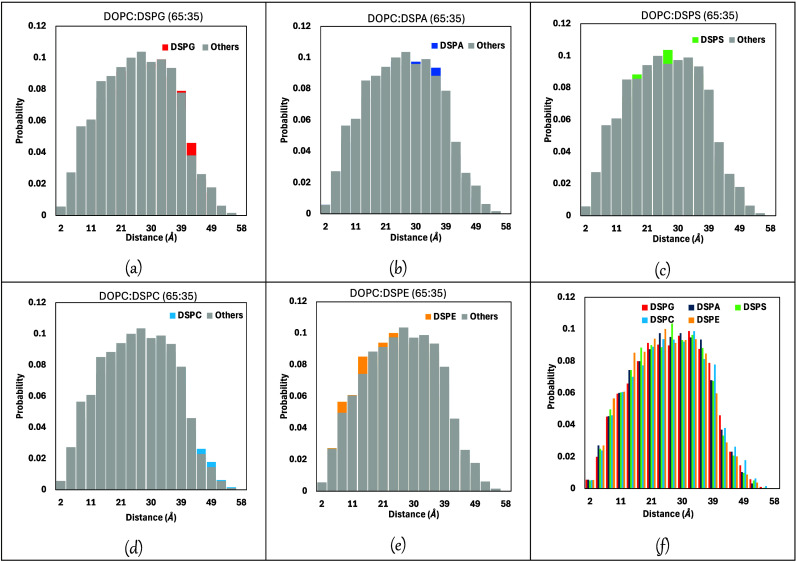
(a–e) Probability histograms of distances between P–P atoms of secondary lipids in each of the 65:35 mixtures considered at a surface tension of γ = 0 mN/m and how they compare against equivalent results for the other mixtures. For example, red bars in Figure 10a illustrate the distances where the LSD between DSPG molecules in a mixture with DOPC have higher probabilities compared to equivalent results for DSPA, DSPS, DSPC, and DSPE mixtures (in gray). (f) LSD profiles for secondary lipids were overlaid all together.

Lipid spacing distribution (LSD) is a representation of the spatial arrangement of lipid molecules within a membrane or lipid bilayer. The LSD between secondary lipids in 65:35 mixtures at a surface tension of γ = 0 mN/m are shown in Figure 10; equivalent results at all surface tensions considered (γ = −7, 0, 7, and 15 mN/m) are shown in Figure S5 (Supporting Information) for 65:35 systems and in Figures S6 and S7 for the 75:25 and 85:15 mixtures. These results are presented as probability distributions of the distances between the phosphorus atoms of the secondary lipids and how they compare against equivalent results for the other secondary lipids studied. For example, red bars in Figure 10a illustrate the probability distribution of distances between DSPG molecules in a 65:35 mixture, overlaid with equivalent results for DSPA, DSPS, DSPC, and DSPE presented in gray. Therefore, the results shown in Figure 10 allow us to visualize the distances where the LSD between a given secondary lipid is higher, compared to equivalent results obtained for the other four 65:35 mixtures. All LSD profiles for secondary lipids are ascribed to Figure 10f.

From Figure 10a, it can be observed that P–P distances between secondary lipids in DOPC:DSPG mixtures (which had the smallest values of *K*_*A*_, Figure 1) have higher probabilities in the 30–45 Å range, which, in general, is larger compared to the distances where the red bars are observed for the other lipid mixtures. Interestingly, the results shown in Figures 4–7 show that the DSPG–DSPG interactions are the weakest (i.e., more positive energy values or less negative energies) among all 65:35 mixtures studied. Conversely, for DOPC:DSPE systems, which had the largest *K*_*A*_ (Figure 1), the yellow bars for DSPE–DSPE distances are observed in the 5–25 Å range (Figure 10e), smaller compared with the distances obtained for the other lipid mixtures. These observations correlate to the results shown in Figures 4–7, which indicate that the DSPE–DSPE energies are the strongest (i.e., more negative) among all 65:35 mixtures. Light blue bars for DSPC systems tend to be in the longer range of distances (Figure 10d), which seems to correlate well with the observation that the DSPC–DSPC interactions were the second weakest among all 65:35 systems (Figure 7a). However, colored bars for DSPA and DSPS mixtures tend to be in the short to middle range of distances, which do not seem to correlate with the results presented in Figure 7a, where the DSPA–DSPA energies are stronger (less positive) compared to those of the DSPS–DSPS interactions. In general, these observations also hold for the histograms shown for the 65:35 mixtures at the other values of surface tensions examined (Figure S5) and for the 75:25 and 85:15 mixtures (Figures S6 and S7); however, as the mole fraction of secondary lipids becomes smaller, there is more variability in the distances where the red bars are observed for the different systems. More insights about the local distribution of lipids in our binary mixtures can be obtained from coarse-grained (CG) simulations, which allowed us to model systems with larger *x*–*y* areas (∼30 × 30 nm^2^) compared to the AA simulations (∼8 × 8 to ∼10 × 10 nm^2^). CG simulation results are discussed in the next section.

### Coarse-Grained (CG) Simulations

**Figure 11:**
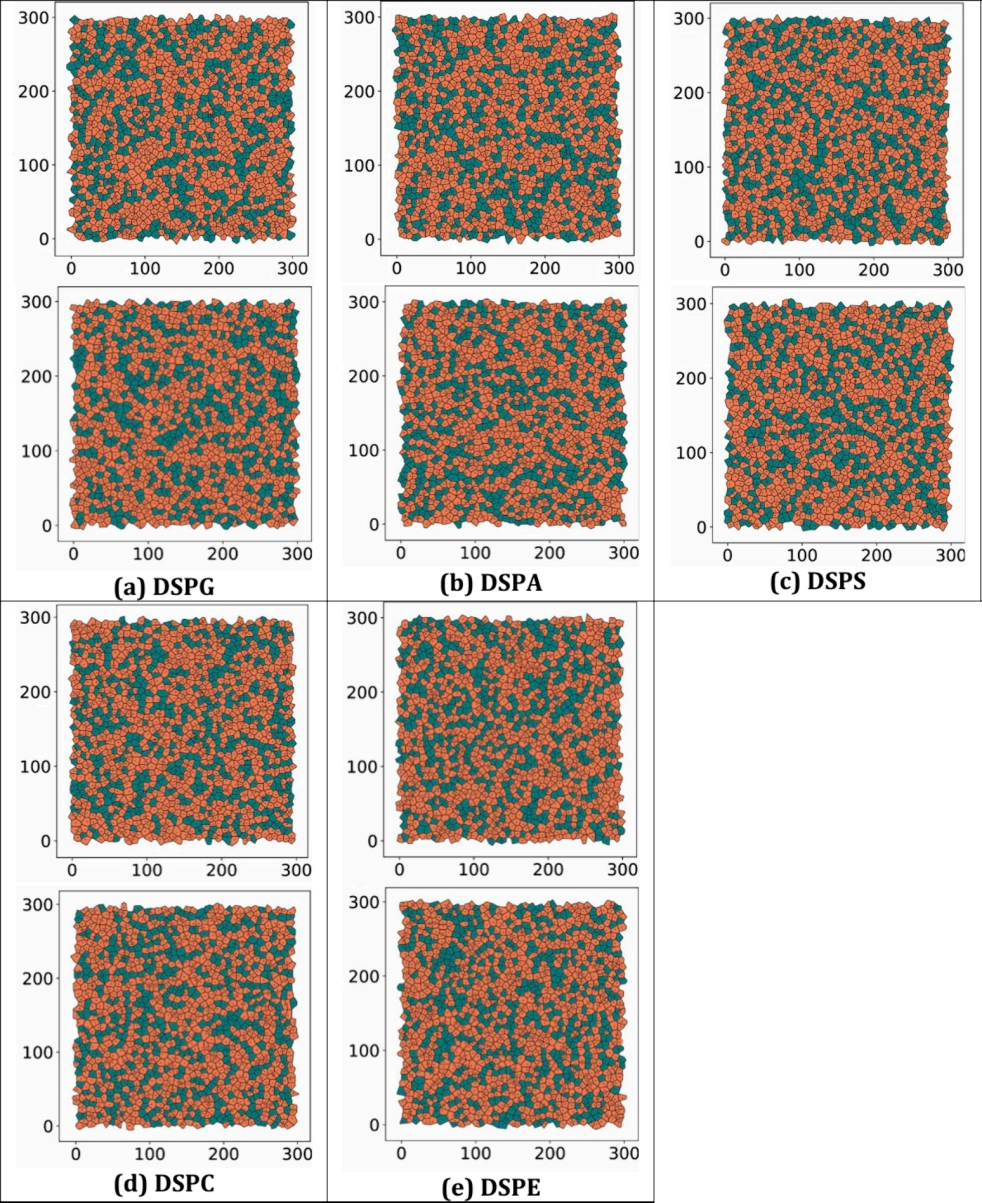
Representative Voronoi diagrams for the top and bottom bilayer leaflets of binary lipid mixtures of 65 mol % DOPC (shown in orange) with the different secondary lipids: (a) DSPG, (b) DSPA, (c) DSPS, (d) DSPC and (e) DSPE (in green), from CG simulations. In all plots, the x-y axes have units of Å.

We performed CG simulations with the Martini force field for pure DOPC bilayers and for our five binary mixtures of lipids, all with 65 mol % DOPC. Voronoi diagrams for the top and bottom bilayer leaflets of these mixtures are presented in Figure 11, which show that in all cases DOPC and the secondary lipids are well mixed. No evidence of inhomogeneities, component segregation, or formation of nanodomains can be observed in these Voronoi diagrams. These observations are similar to what was found in our previous study,^31^ where DOPC was mixed with lipids having the same headgroup but shorter saturated acyl tails. Monticelli et al.^29^ argued that, for homogeneous lipid mixtures, the bending modulus *k*_*C*_ is similar to the composition-weighted average of the *k*_*C*_ values of the individual components; however, in the inhomogeneous or phase separated mixtures that they studied, the observed *k*_*C*_ is closer to the value of the softer components, but here *k*_*C*_ varies with position according to the local composition. Special approaches might be needed to study the mechanical properties of these lipid nanodomains.^83^ As these mixture inhomogeneities can affect measured mechanical properties, detecting them in simulations is crucial as conventional experimental measurements may lack resolution to observe composition inhomogeneities; this observation highlights the importance of simulations as “molecular microscopes” that can complement and guide experimental studies. We also note that lipid nanodomains have been reported for mixtures of the lipids considered here with cholesterol.^84,85^

From the results shown in Figure 11, we computed the distribution of areas of the Voronoi cells corresponding to the primary and secondary lipids, which can provide information about local variations in the area per lipid value in our bilayer systems. The area distributions are plotted in the histograms shown in Figure S8 (Supporting Information). Mean (average) and median values of these area distributions are listed in Table S6. Voronoi cell areas span from 30 to 110 A^2^ for all lipids and show a right-skewed distribution for all systems, where the DOPC histograms are, in general, less smooth compared to the distributions found for the secondary lipids. The average and median values of Voronoi cell area for the primary lipid experience slight reductions when going from 100 to 65 mol % DOPC, suggesting that increasing the concentration of secondary lipids does not lead to important changes in lipid packing. In all mixtures the average Voronoi cell area is ∼65 Å^2^, similar to the values found in the mixtures of our previous study;^31^ however, the DSPG mixture (which had the smallest *K*_*A*_ among our systems, Figure 1) has slightly smaller average and median areas compared to the other 65:35 mixtures.

We calculated the percentage of mixed contacts^29^ as a quantitative measure of homogeneity in our CG mixtures
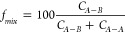
5where *C*_*A*–*A*_ and *C*_*A*–*B*_ are the number of contacts between the same type and different type of lipids, respectively. Lipids are considered to be in contact if the distance between the PO_4_ beads is smaller than 1.1 nm. *f*_*mix*_ quantifies the proportion of contacts between primary and secondary lipids relative to the total number of contacts. These results are shown in Table 4; we also ran simulations for slightly larger CG systems [labeled (*) in Table S1], with equivalent results shown in Table S7 (Supporting Information). Equivalent results for our AA systems are presented in Table S8 (Supporting Information). No significant differences are observed between CG and AA results for mixed contacts (Tables 4 and S8); however, the uncertainties in AA simulations are larger due to the smaller system sizes. The results shown in Tables 4, S7, and S8 are very similar to each other and indicate that, for all systems, *f*_*mix*_ is very close to the average mole % of the secondary lipids (35%). However, the observed variations from the average composition are larger than the values reported in our previous study,^31^ for mixtures of DOPC with saturated lipids with the same headgroup but shorter tail lengths. *f*_*mix*_ is the largest in the 65:35 DOPC:DSPG system (the softest among our systems) and is the smallest for the DOPC:DSPE system, which had the largest *K*_*A*_ among the mixtures examined.

**Table 4:** Percentage of Mixed Contacts in the CG Systems^a^

**System**	***f***_***mix***_
DOPC–DSPG	37.29 ± 0.60
DOPC–DSPA	37.04 ± 0.60
DOPC–DSPS	36.43 ± 0.71
DOPC–DSPC	35.18 ± 0.65
DOPC–DSPE	32.64 ± 0.76

a Equivalent results for slightly larger CG systems [labeled (*) in Table S1] are presented in Table S7. Similar results for our AA systems are shown in Table S8.

We also computed the number and type of neighboring lipids around each lipid species,^86^ as another way to quantify homogeneity in our CG systems. Two lipids are considered neighbors if the distance between their GL1 bead (the first bead in the lipid tail, Table 2) is smaller than or equal to 1.5 nm.^86^ These results are shown in Table 5 and in Table S9 for equivalent CG systems with slightly larger sizes. Equivalent results for our AA simulations are reported in Table S10; no significant differences are observed between the results for AA and CG systems, suggesting again that a similar lipid mixing behavior is observed between both types of systems. Likewise, both sets of CG results (Tables 5 and S9) are in good agreement with the reported uncertainty values. All local compositions of neighboring lipids are quite similar to the overall composition of our systems, 65:35, suggesting again that our binary lipid mixtures are well mixed. Interestingly, the larger deviations between local and overall compositions are observed for DOPC–DSPE, the stiffest system among all mixtures examined, whereas, for the softest DOPC–DSPG systems, the local and global compositions are quite similar.

**Table 5:** Number and Type of Neighboring Lipids in the CG Systems^a^

**Type of Center Lipid**	**Type of Neighbor Lipids**	
	**DOPC**	**DSPG**
**DOPC**	5.14 ± 0.18 (65.7%)	2.68 ± 0.21 (34.3%)
**DSPG**	5.35 ± 0.27 (64.8%)	2.90 ± 0.19 (35.2%)
	**DOPC**	**DSPA**
**DOPC**	5.29 ± 0.19 (66.9%)	2.62 ± 0.10 (33.1%)
**DSPA**	5.25 ± 0.20 (63.6%)	3.00 ± 0.23 (36.4%)
	**DOPC**	**DSPS**
**DOPC**	5.29 ± 0.08 (66.8%)	2.63 ± 0.06 (33.2%)
**DSPS**	5.25 ± 0.12 (65.0%)	2.83 ± 0.15 (35.0%)
	**DOPC**	**DSPC**
**DOPC**	5.37 ± 0.35 (67.9%)	2.54 ± 0.09 (32.1%)
**DSPC**	5.09 ± 0.22 (62.8%)	3.02 ± 0.22 (37.2%)
	**DOPC**	**DSPE**
**DOPC**	5.58 ± 0.18 (69.7%)	2.43 ± 0.18 (30.3%)
**DSPE**	4.85 ± 0.27 (58.8%)	3.40 ± 0.18 (41.2%)

a For example, in a DOPC–DSPG system, a DOPC molecule is surrounded by an average of 5.14 DOPC and 2.68 DSPG molecules, corresponding respectively to 65.7% and 34.3% of the average number of neighboring lipids. Equivalent results for slightly larger CG systems [labeled (*) in Table S1] are presented in Table S9. Similar results for our AA systems are shown in Table S10.

### In Vitro Cellular Internalization of Liposomes

**Figure 12:**
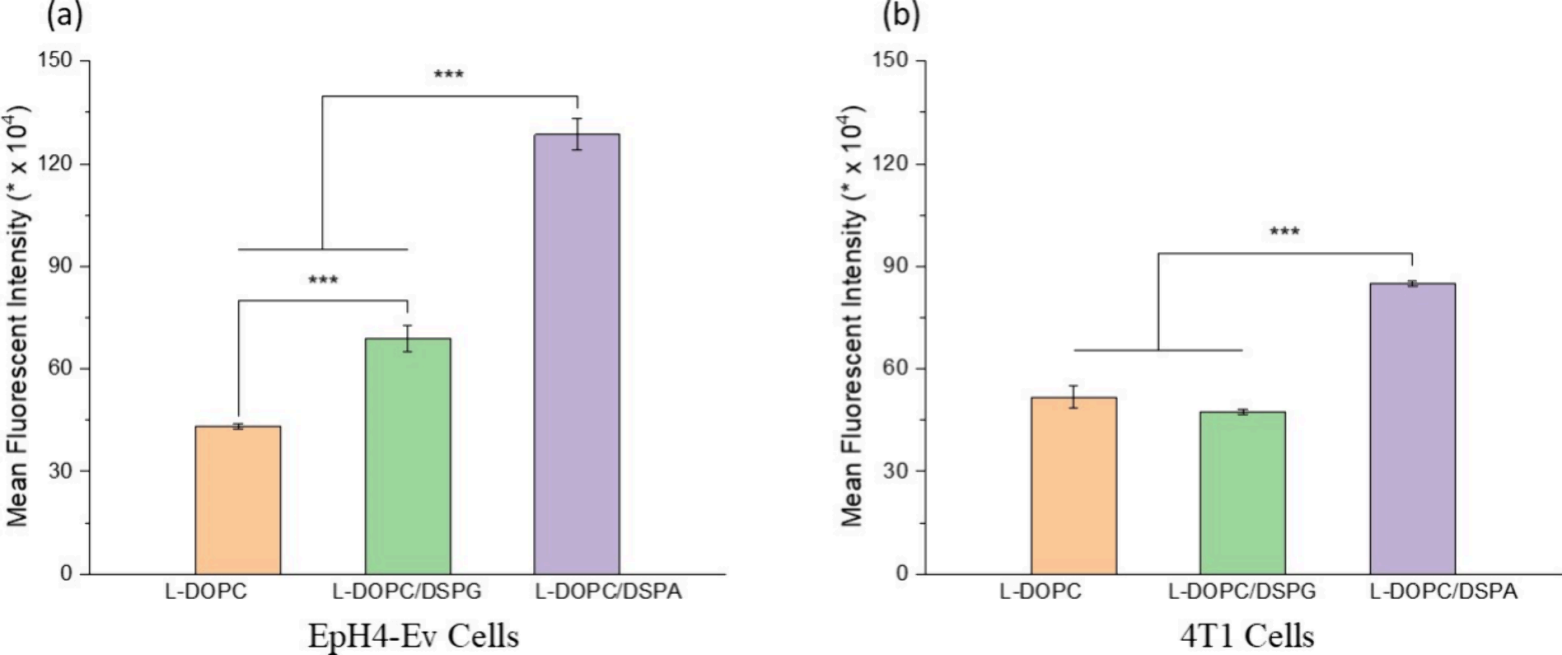
In vitro cellular internalization of L-DOPC, L-DOPC/DSPG, and L-DOPC/DSPA in (a) mouse normal epithelial cells (EpH4-Ev) and (b) mouse MBC cells (4T1). All values are presented as the mean ± standard deviation. For all measurements, *n* = 3. Statistical analysis was done using ANOVA with post hoc Tukey test. Significance is indicated on the figure for each experiment as follows: * *p* < 0.05, ** *p* < 0.01, *** *p* < 0.001.

Correlating computational data with experimental results may help engineer drug delivery systems that efficiently target tumor tissue and favorably interact with cancer cells. Techniques such as micropipette aspiration^69−71^ and nanoindentation^87−90^ can be used to measure the mechanical properties of liposomes made with the lipid mixtures identified in this study, but we currently lack access to the necessary instrumentation to perform these experiments. To gain insights on how the different lipid headgroups may affect cell–nanoparticle interactions, we synthesized three liposomal formulations using DOPC, DSPG, and DSPA, where L-DOPC contained only DOPC while L-DOPC/DSPG and L-DOPC/DSPA contained 15 mol % of DSPG and DSPA, respectively. Then, we evaluated the cellular internalization of these formulations in normal epithelial cells (EpH-4Ev) and metastatic breast cancer cells (MBC, 4T1). Among the systems studied through simulations, DOPC:DSPG and DOPC:DSPA were the softest, having the largest differences in area compressibility modulus *K*_*A*_ compared to pure DOPC (Figure 1 and Table S2). Liposome charge can greatly impact uptake by cells; however, as both DSPA and DSPG are negatively charged, any differences in cell uptake for both normal and cancer cells can be attributed to changes in the mechanical properties. The results showed that L-DOPC/DSPA had approximately 3- and 2-times higher internalization in EpH4-Ev and 4T1 cells, respectively, compared with L-DOPC (Figure 12a,b). In addition, the uptake of L-DOPC/DSPA was significantly higher than that of L-DOPC/DSPG in both cell lines. The internalization of L-DOPC/DSPG in normal epithelial cells EpH-4Ev was almost 2-fold compared to that of L-DOPC, but both liposomes had similar uptakes by 4T1 cancer cells (Figure 12a,b).

We have previously shown^31,32^ that the addition of 25–35 mol % of the short-chain phospholipid DHPC to DOPC liposomes decreased the *K*_*A*_ values of their lipid membranes by approximately 10% compared to pure DOPC liposomes. These results indicated a less rigid lipid bilayer in the case of the DHPC-incorporated liposomes, which were confirmed experimentally by micropipette aspiration measurement that showed a 14–33% decrease in the stretching moduli of the mixed-lipid liposomes.^31,32^ These softer liposomes showed significantly higher internalization in healthy and cancer cells compared with the pure DOPC liposomes.^31,32^ In this study, MD data showed that the 85:15 systems of DSPG and DSPA both had approximately 13% lower *K*_*A*_, indicating less rigid lipid bilayers, compared with those of the pure DOPC system (Figure 1 and Table S2). This observation might have contributed to the higher cellular internalization of L-DOPC/DSPA and L-DOPC/DSPG compared to L-DOPC (Figure 12a,b).

On the other hand, MD data showed that the *K*_*A*_ values for the 85:15 systems of DSPG and DSPA were not different within the reported uncertainties (Figure 1 and Table S2). Therefore, we expect that other factors might have contributed to the differences between their respective cellular uptakes observed experimentally. The data shown in Table 3 indicates that the L-DOPC/DSPA formulations had the smallest diffusion coefficients and largest effective diameters, followed by L-DOPC/DSPG and L-DOPC. Although the data showed that both systems had negative ESP values, their respective charge distributions were different (Figure 2b,c). This observation might have led to the formation of liposomes with a more negative ζ-potential in the case of L-DOPC/DSPA compared with L-DOPC/DSPG, as shown in Table 3. We presume that the more negative charge on the surface of L-DOPC/DSPA might have led to increased interactions with ions in the surrounding solution, in agreement with the interaction energy calculations previously discussed (Figure 8). Moreover, the increased overall charge on the surface of L-DOPC/DSPA could have increased electrostatic interactions with lipid headgroups and protein domains that carry opposite charges in the cell membrane. This might have facilitated the cellular internalization of L-DOPC/DSPA compared with L-DOPC/DSPG and L-DOPC. These differences combined might have contributed to the different levels of cellular uptake of these formulations.

## Conclusions

We performed molecular dynamics simulations of binary mixtures of the phospholipid DOPC (which has two acyl tails, each with 18 carbon atoms and a double bond between carbons 9 and 10) as the primary component, with secondary lipids (DSPG, DSPA, DSPC, DSPS, or DSPE) that also have 18 carbon atoms in their tails, but all bonds in their acyl chains are saturated and have different headgroups than DOPC. Our all-atom simulation results indicate that the DOPC:DSPG system with 65:35 molar ratio is the softest, having an area compressibility modulus *K*_*A*_ that is about 22% smaller than the value obtained for a pure DOPC bilayer. Increases in the mole % of DOPC lead to increases in *K*_*A*_, where at any given composition the *K*_*A*_ trend among the five systems is DSPG < DSPA < DSPS < DSPC < DSPE, except for the 95:5 systems where all have *K*_*A*_ that are statistically similar to the value observed for a pure DOPC bilayer. Incorporation of DSPE into DOPC bilayers did not alter the stiffness within the range of concentrations examined. Lipid–lipid interactions tend to be weaker in mixtures with DSPG and stronger in mixtures containing DSPE, and thus, the distances between DSPG lipids are larger compared to the DSPE–DSPE distances. The head and phosphate groups of the secondary lipids DSPG, DSPA, and DSPS interact strongly with Na^+^ and Cl^–^ ions, and they have electrostatic potential (ESP) surfaces consisting entirely of negative values, whereas DSPC and DSPE have weaker interactions with ions and their ESP surfaces range between positive and negative values. These observations suggest that the ion concentration can alter the mechanical properties of mixed lipid bilayers. Addition of the secondary lipids leads to increases in the order parameters of the acyl chains of DOPC, which are larger than the values observed in a pure DOPC bilayer. Furthermore, the acyl tails of the secondary lipids tend to have larger order parameter values in systems with DSPG than those in bilayers containing DSPE at any given composition. Simulations with coarse-grained models show no evidence of phase separation or inhomogeneity in the examined 65:35 mixtures. In general, the local compositions in all mixtures are similar to the average overall composition, with the stiffest DOPC:DSPE systems having the largest local variations from the overall molar ratio.

We synthesized three liposomal formulations, where L-DOPC contained only DOPC while L-DOPC/DSPG and L-DOPC/DSPA contained 15 mol % of DSPG and DSPA, respectively. L-DOPC/DSPA had approximately 3- and 2-times higher internalization by normal epithelial (EpH4-Ev) and metastatic breast cancer (4T1) cells, respectively, compared with L-DOPC. The uptake of L-DOPC/DSPG by normal epithelial cells was almost 2-fold compared to L-DOPC, but both liposomes had similar uptakes by cancer cells. As L-DOPC/DSPG and L-DOPC/DSPA have similar *K*_*A*_ values (both 12–13% smaller compared to L-DOPC), we presumed that higher negative charges in L-DOPC/DSPA, possibly in combination with differences in effective liposome diameters and diffusivities, contributed to these cell uptake observations. Taken together, our simulations indicate that the maximum reduction in *K*_*A*_ from the value observed in a pure DOPC system is relatively modest in our systems (a maximum of ∼22% in a 65:35 DOPC:DSPG mixture). However, these results are promising and suggest that systematically exploring the phase space of binary mixtures of lipids with different molecular structures will result in fundamentally understanding the factors that govern the elasticity and rigidity of lipid bilayers. Such fundamental knowledge will allow us to develop customized liposome formulations with the desired mechanical properties for different applications. Nevertheless, our experimental results highlight the fact that liposome cell uptakes have a complex dependence on the particles’ mechanical properties, surface charge, and size, among other possible factors. In future studies, we plan to identify additional promising lipid mixtures through simulation studies, and as we did in our previous studies,^31,32^ we will collaborate with scientists who have the equipment and expertise required to carry out micropipette aspiration measurements, to corroborate our findings. Future studies can also focus on binary DOPC:DOPx lipid mixtures, i.e., where the secondary lipids also have a double bond in their acyl tails and the same structure as DOPC except their headgroup; such studies would help deconvolute the role that lipid head and tail groups play in modulating the mechanical properties of the resulting liposomes.

## Supporting Information Available

Details of model systems (Table S1); numerical values for area compressibility modulus, lipid–lipid partial and total interaction energies, interaction energies between head and phosphate groups of secondary lipids with salt ions, from all-atom simulations (Tables S2–S5); coarse-grained simulation results for mean and median Voronoi cell areas, percentage of mixed contacts and number and type of neighboring lipids (Tables S6–S10); area per lipid results (Figure S1); order parameter plots (Figures S2–S4); probability histograms of distances between P atoms of secondary lipids (Figures S5–S7); histograms of Voronoi cell areas (Figure S8) (PDF)
