# Supplementary material for: Effects of Lipid Headgroups on the Mechanical Properties and In Vitro Cellular Internalization of Liposomes
Source: Langmuir. 2025 Jan 21;41(4):2600–18. doi: 10.1021/acs.langmuir.4c04363 (PMC11803717; doi:10.1021/acs.langmuir.4c04363)
Supplement: Supplementary file 1 — la4c04363_si_001.pdf [file la4c04363_si_001.pdf]

Effects of Lipid Headgroups on the Mechanical Properties and In Vitro Cellular Internalization of Liposomes

Supporting Information

Jiaming Xu,[1] Stephen Adepoju,[1] Simran Pandey,[1] Jimena Pérez Tetuán,[2] Mary Williams,[1] Rudolf G. Abdelmessih,[1] Debra T. Auguste[1,2] and Francisco R. Hung[1,*]

[1] Department of Chemical Engineering, Northeastern University, Boston, MA 02115, USA

[2] Department of Bioengineering, Northeastern University, Boston, MA 02115, USA

* Corresponding author. E-mail: f.hung@northeastern.edu.

Table S1. Details of lipid bilayer systems investigated in this work. Systems labeled (*) had larger sizes and were considered for corroboration purposes.

All-atom (AA)			Coarse-grained (CG)		
**System**	N_{DOPC}:$N_{sec.lipids}$	mol_{DOPC}%	**System**	N_{DOPC}:$N_{sec.lipids}$	mol_{DOPC}%
**DOPC:DSPA**	130:70	65	**DOPC:DSPA**	1794:966	65
**DOPC:DSPA (*)**	187:101	65	**DOPC:DSPC**	1742:938	65
**DOPC:DSPA**	150:50	75	**DOPC:DSPC (*)**	1976:1064	65
**DOPC:DSPA**	170:30	85	**DOPC:DSPE**	1794:966	65
**DOPC:DSPA**	190:10	95	**DOPC:DSPE (*)**	1976:1064	65
**DOPC:DSPC**	130:70	65	**DOPC:DSPG**	1768:952	65
**DOPC:DSPC**	150:50	75	**DOPC:DSPG (*)**	1976:1064	65
**DOPC:DSPC**	170:30	85	**DOPC:DSPG (*)**	1768:952	65
**DOPC:DSPC**	190:10	95	**DOPC:DSPS**	1768:952	65
**DOPC:DSPE**	130:70	65	**DOPC:DSPS (*)**	1976:1064	65
**DOPC:DSPE (*)**	187:101	65			
**DOPC:DSPE**	150:50	75			
**DOPC:DSPE**	170:30	85			
**DOPC:DSPE**	190:10	95			
**DOPC:DSPG**	130:70	65			
**DOPC:DSPG (*)**	187:101	65			
**DOPC:DSPG**	150:50	75			
**DOPC:DSPG**	170:30	85			
**DOPC:DSPG**	190:10	95			
**DOPC:DSPS**	130:70	65			
**DOPC:DSPS (*)**	187:101	65			
**DOPC:DSPS**	150:50	75			
**DOPC:DSPS**	170:30	85			
**DOPC:DSPS**	190:10	95			
**Pure DSPA**	0:200	0			
**Pure DSPC**	0:200	0			
**Pure DSPE**	0:200	0			
**Pure DSPG**	0:200	0			
**Pure DSPS**	0:200	0			

Table S2. Area compressibility modulus K_A of our bilayer systems as determined from all-atom MD simulations. Molar percentages of primary and secondary lipid component are shown in parentheses. Systems labeled (*) had larger sizes and were considered for corroboration purposes. The columns labeled 'Difference' display the percentage change in K_A compared to the value obtained for the pure DOPC bilayer. A two-sample t-test was performed to assess the difference between each considered mixed system and their corresponding pure bilayer. The t-test was conducted at a 95% confidence level with degrees of freedom computed as $df = n_1 + n_2 - 2$. As all K_A values were determined from simulations at four values of surface tension, $n_1 = n_2 = 4$, giving $df = 6$. From the table of critical values of t for two-tailed tests,[1] we obtain $t = 2.447$. Therefore, systems with absolute values of t larger than 2.447 (not shaded) indicate mixed bilayers with K_A values that are statistically different from the values observed in pure DOPC systems.

Bilayer composition	K_A (mN/m)	Difference	*t*-score	*p*-value
**DOPC (100)**	245.8 ± 9.9	-	-	-
**DOPC:DSPG (65:35)**	192.7 ± 10.8	21.60%	-7.24	0.0004
**DOPC:DSPG (65:35) (*)**	200.1 ± 7.4	18.59%	-7.41	0.0003
**DOPC:DSPA (65:35)**	201.5 ± 7.0	18.02%	-7.31	0.0003
**DOPC:DSPA (65:35) (*)**	189.0 ± 8.1	23.11%	-8.89	0.0001
**DOPC:DSPS (65:35)**	203.1 ± 3.6	17.37%	-8.12	0.0002
**DOPC:DSPS (65:35) (*)**	206.5 ± 21.8	15.99%	-3.28	0.0168
**DOPC:DSPC (65:35)**	219.6 ± 8.2	10.66%	-4.08	0.0065
**DOPC:DSPE (65:35)**	243.7 ± 11.4	0.85%	-0.28	0.7905
**DOPC:DSPE (65:35) (*)**	214.6 ± 12.7	12.69%	-3.38	0.0082
**DOPC:DSPG (75:25)**	200.1 ± 7.0	18.59%	-7.55	0.0003
**DOPC:DSPA (75:25)**	212.9 ± 5.8	13.38%	-5.72	0.0012
**DOPC:DSPS (75:25)**	210.6 ± 5.5	14.32%	-6.20	0.0008
**DOPC:DSPC (75:25)**	224.2 ± 7.4	8.79%	-3.51	0.0127
**DOPC:DSPE (75:25)**	234.3 ± 10.4	4.68%	-1.60	0.1615
**DOPC:DSPG (85:15)**	214.2 ± 9.3	12.86%	-4.66	0.0035
**DOPC:DSPA (85:15)**	216.4 ± 7.8	11.96%	-4.66	0.0035
**DOPC:DSPS (85:15)**	215.1 ± 5.7	12.49%	-5.38	0.0017
**DOPC:DSPC (85:15)**	232.3 ± 12.1	5.49%	-1.72	0.1355
**DOPC:DSPE (85:15)**	236.6 ± 11.9	3.74%	-1.19	0.2786
**DOPC:DSPG (95:5)**	253.5 ± 10.5	3.13%	1.06	0.3307
**DOPC:DSPA (95:5)**	251.5 ± 8.3	2.32%	0.89	0.4094
**DOPC:DSPS (95:5)**	232.0 ± 7.3	5.61%	-2.25	0.0659
**DOPC:DSPC (95:5)**	254.3 ± 15.7	3.46%	0.91	0.3958
**DOPC:DSPE (95:5)**	226.2 ± 7.4	7.97%	-3.18	0.0192

Table S3. Numerical values and uncertainties of partial interaction energies, in kcal/mol (cfg. Fig. 3b). DS-DS, DOPC-DOPC and DS-DOPC indicate interactions secondary lipid-secondary lipid, DOPC-DOPC and secondary lipid-DOPC, respectively.

System	DS-DS	Std. Dev	DOPC-DOPC	Std. Dev.	DS-DOPC	Std. Dev.	Partial Energy	Std. Dev.
**DOPC:DSPA (65:35)**	2044.9	12.5	-13649.2	19.6	-5171.1	16.2	-16775.5	48.3
**DOPC:DSPA (75:25)**	1498.1	11.3	-17224.3	22.8	-4318.9	17.1	-20045.1	51.3
**DOPC:DSPA (85:15)**	966.9	8.2	-20470.4	25.8	-2946.1	12.2	-22449.6	46.2
**DOPC:DSPA (95:5)**	324.5	4.6	-24547.5	26.4	-1130.1	7.4	-25353.1	38.4
**DOPC:DSPC (65:35)**	-4391.5	12.7	-13183.0	21.8	-4923.4	16.8	-22497.9	51.3
**DOPC:DSPC (75:25)**	-2560.8	10.9	-16693.1	23.2	-3958.9	14.6	-23212.8	48.7
**DOPC:DSPC (85:15)**	-1120.9	8.6	-19849.5	25.4	-2681.3	12.0	-23651.6	46.1
**DOPC:DSPC (95:5)**	-332.6	4.4	-24037.2	26.9	-956.8	7.5	-25326.6	38.7
**DOPC:DSPE (65:35)**	-7081.7	12.9	-15113.1	21.3	-5584.9	14.9	-27779.7	49.2
**DOPC:DSPE (75:25)**	-3181.4	12.3	-17078.8	23.7	-4865.0	14.9	-25125.2	51.0
**DOPC:DSPE (85:15)**	-949.9	8.4	-20439.1	24.9	-3435.3	11.5	-24824.3	44.7
**DOPC:DSPE (95:5)**	-145.2	4.2	-23961.1	26.3	-1304.2	7.1	-25410.5	37.7
**DOPC:DSPG (65:35)**	2518.1	13.6	-12601.2	21.6	-5293.0	16.9	-15376.2	52.1
**DOPC:DSPG (75:25)**	2005.7	11.0	-15689.2	25.0	-4496.9	15.3	-18180.4	51.3
**DOPC:DSPG (85:15)**	1213.1	9.8	-20023.2	26.9	-3084.1	11.1	-21894.3	47.8
**DOPC:DSPG (95:5)**	442.4	4.3	-23897.2	27.0	-1146.9	7.4	-24601.7	38.7
**DOPC:DSPS (65:35)**	1197.1	14.2	-14032.2	23.1	-6320.4	17.3	-19155.6	54.6
**DOPC:DSPS (75:25)**	1059.7	10.9	-17082.6	24.3	-5264.2	14.3	-21287.1	49.5
**DOPC:DSPS (85:15)**	944.2	7.6	-20470.1	25.1	-3691.7	12.1	-23217.7	44.8
**DOPC:DSPS (95:5)**	331.3	4.0	-24317.5	26.9	-1357.8	7.0	-25344.0	37.9

Table S4. Numerical values and uncertainties of total interaction energies, in kcal/mol (cfg. Fig. 3b). DS-DS, DOPC-DOPC and DS-DOPC indicate interactions secondary lipid-secondary lipid, DOPC-DOPC and secondary lipid-DOPC, respectively.

System	DS-DS	Std. Dev.	DOPC-DOPC	Std. Dev.	DS-DOPC	Std. Dev.	Total Energy	Std. Dev.
**DOPC:DSPA (65:35)**	8015.7	12.5	22260.6	19.6	-5171.1	16.2	25105.1	89.5
**DOPC:DSPA (75:25)**	5821.1	11.3	24827.1	22.8	-4318.9	17.1	26329.3	89.4
**DOPC:DSPA (85:15)**	3410.5	8.2	27065.7	25.8	-2946.1	12.2	27530.1	81.6
**DOPC:DSPA (95:5)**	1200.9	4.6	29241.2	26.4	-1130.1	7.4	29311.9	71.4
**DOPC:DSPC (65:35)**	12534.0	12.7	21828.8	21.8	-4923.4	16.8	29439.4	91.1
**DOPC:DSPC (75:25)**	9223.4	10.9	24451.3	23.2	-3958.9	14.6	29715.8	86.5
**DOPC:DSPC (85:15)**	5724.3	8.6	27053.5	25.4	-2681.3	12.0	30096.6	80.6
**DOPC:DSPC (95:5)**	1980.8	4.4	29073.0	26.9	-956.8	7.5	30097.0	70.0
**DOPC:DSPE (65:35)**	11181.1	12.9	22117.1	21.3	-5584.9	14.9	27713.3	88.5
**DOPC:DSPE (75:25)**	8544.9	12.3	24794.3	23.7	-4865.0	14.9	28474.1	87.6
**DOPC:DSPE (85:15)**	5454.6	8.4	27242.5	24.9	-3435.3	11.5	29261.9	81.9
**DOPC:DSPE (95:5)**	1886.8	4.2	29381.0	26.3	-1304.2	7.2	29963.6	69.9
**DOPC:DSPG (65:35)**	17323.0	13.6	22193.7	21.6	-5293.0	16.9	34223.7	92.4
**DOPC:DSPG (75:25)**	12242.5	11.0	24825.6	25.0	-4496.9	15.3	32571.2	86.0
**DOPC:DSPG (85:15)**	7464.9	9.8	27147.9	26.9	-3084.1	11.1	31528.7	83.0
**DOPC:DSPG (95:5)**	2514.6	4.3	29302.2	27.0	-1146.9	7.4	30669.9	72.9
**DOPC:DSPS (65:35)**	11592.7	14.2	22167.5	23.1	-6320.4	17.3	27439.8	90.7
**DOPC:DSPS (75:25)**	8475.7	10.9	24634.9	24.3	-5264.2	14.3	27846.5	87.0
**DOPC:DSPS (85:15)**	5123.1	7.6	27233.9	25.1	-3691.7	12.1	28665.3	83.9
**DOPC:DSPS (95:5)**	1780.1	4.0	29186.0	26.9	-1357.8	7.0	29608.4	74.4

Table S5. Interaction energy (kcal/mol) between salt ions and head + phosphate groups of the secondary lipids in the binary mixtures studied. As DSPA 'lacks' a headgroup (it is only a hydrogen atom, Table 1), only the ion-phosphate interactions are presented.

	**Secondary lipid**	**65% DOPC**	**75% DOPC**	**85% DOPC**	**95% DOPC**
**HEAD**	**DSPA**	-	-	-	-
	**DSPC**	-6.10 ± 0.06	-4.64 ± 0.06	-6.48 ± 0.03	-2.69 ± 0.03
	**DSPE**	-2.95 ± 0.03	-2.38 ± 0.05	-2.05 ± 0.01	-4.35 ± 0.02
	**DSPG**	-47.4 ± 0.60	-31.6 ± 0.4	-22.2 ± 0.2	-13.1 ± 0.2
	**DSPS**	-51.9 ± 0.4	-36.6 ± 0.5	-22.5 ± 0.3	-16.99 ± 0.02
**PHOS**	**DSPA**	-57.3 ± 1.1	-44.0 ± 0.9	-29.9 ± 0.5	-21.1 ± 0.3
	**DSPC**	2.44 ± 0.04	3.08 ± 0.06	3.73 ± 0.05	7.38 ± 0.03
	**DSPE**	1.690 ± 0.009	0.96 ± 0.02	0.32 ± 0.01	1.86 ± 0.01
	**DSPG**	1.060 ± 0.001	0.370 ± 0.004	0.12 ± 0.01	0.22 ± 0.01
	**DSPS**	-11.1 ± 0.08	-10.9 ± 0.1	-8.3 ± 0.1	-6.56 ± 0.01

Table S6. Mean and median Voronoi cell area ($\mathring{A}^2$) of DOPC and secondary lipids in different 65:35 CG systems.

	**Mean**		**Median**	
**System**	**DOPC**	**Secondary Lipid**	**DOPC**	**Secondary Lipid**
**DOPC-DSPG**	64.62	64.68	63.20	63.08
**DOPC-DSPA**	65.15	65.08	63.61	63.56
**DOPC-DSPS**	65.54	65.54	64.18	64.21
**DOPC-DSPC**	65.68	65.72	64.28	64.38
**DOPC-DSPE**	65.21	65.20	63.73	63.75
**DOPC**	67.58	-	66.06	-

Table S7. Percentage of mixed contacts in the CG systems with slightly larger sizes [labeled (*) in Table S1].

**System**	f_{mix}
DOPC-DSPG	35.94 ± 0.73
DOPC-DSPG	37.80 ± 0.61
DOPC-DSPS	35.06 ± 0.61
DOPC-DSPC	35.47 ± 0.65
DOPC-DSPE	33.79 ± 0.71

Table S8. Percentage of mixed contacts in the AA systems.

**System**	f_{mix}
DOPC-DSPG	38.44 ± 2.31
DOPC-DSPA	40.29 ± 2.12
DOPC-DSPS	38.24 ± 2.26
DOPC-DSPC	36.90 ± 2.43
DOPC-DSPE	35.06 ± 2.05

Table S9. Number and type of neighboring lipids in our CG systems with slightly larger sizes [labeled (*) in Table S1]. For example, in the first DOPC-DSPG system, a DOPC molecule is surrounded by an average of 5.05 DOPC and 2.48 DSPG molecules, corresponding respectively to 67.1% and 32.9% of the average number of neighboring lipids.

Type of center lipid	Type of neighbor lipids	
	**DOPC**	**DSPG**
**DOPC**	5.05 ± 0.12 (67.1%)	2.48 ± 0.27 (32.9%)
**DSPG**	5.45 ± 0.23 (64.6%)	2.99 ± 0.29 (35.4%)
	**DOPC**	**DSPG**
**DOPC**	5.34 ± 0.14 (65.5%)	2.81 ± 0.23 (34.5%)
**DSPG**	5.65 ± 0.11 (66.0%)	2.91 ± 0.21 (34.0%)
	**DOPC**	**DSPS**
**DOPC**	5.39 ± 0.12 (67.1%)	2.64 ± 0.16 (32.9%)
**DSPS**	5.15 ± 0.17 (64.2%)	2.87 ± 0.05 (35.8%)
	**DOPC**	**DSPC**
**DOPC**	5.14 ± 0.15 (67.5%)	2.48 ± 0.13 (32.5%)
**DSPC**	5.09 ± 0.12 (62.0%)	3.12 ± 0.13 (38.0%)
	**DOPC**	**DSPE**
**DOPC**	5.61 ± 0.12 (72.5%)	2.13 ± 0.11 (27.5%)
**DSPE**	5.88 ± 0.21 (64.0%)	3.31 ± 0.22 (36.0%)

Table S10. Number and type of neighboring lipids in our AA systems. For example, in a DOPC-DSPG system, a DOPC molecule is surrounded by an average of 5.18 DOPC and 2.72 DSPG molecules, corresponding respectively to 65.6% and 34.4% of the average number of neighboring lipids.

Type of center lipid	Type of neighbor lipids	
	**DOPC**	**DSPG**
**DOPC**	5.18 ± 0.19 (65.6%)	2.72 ± 0.22 (34.4%)
**DSPG**	5.30 ± 0.28 (64.9%)	2.87 ± 0.20 (35.1%)
	**DOPC**	**DSPA**
**DOPC**	5.32 ± 0.20 (66.8%)	2.65 ± 0.11 (33.2%)
**DSPA**	5.23 ± 0.22 (63.3%)	3.03 ± 0.25 (36.7%)
	**DOPC**	**DSPS**
**DOPC**	5.26 ± 0.09 (66.8%)	2.66 ± 0.07 (33.6%)
**DSPS**	5.22 ± 0.13 (64.6%)	2.86 ± 0.16 (35.4%)
	**DOPC**	**DSPC**
**DOPC**	5.34 ± 0.33 (67.3%)	2.59 ± 0.10 (32.7%)
**DSPC**	5.07 ± 0.23 (62.4%)	3.06 ± 0.24 (37.6%)
	**DOPC**	**DSPE**
**DOPC**	5.60 ± 0.19 (69.4%)	2.47 ± 0.19 (30.6%)
**DSPE**	4.87 ± 0.28 (58.7%)	3.42 ± 0.19 (41.3%)

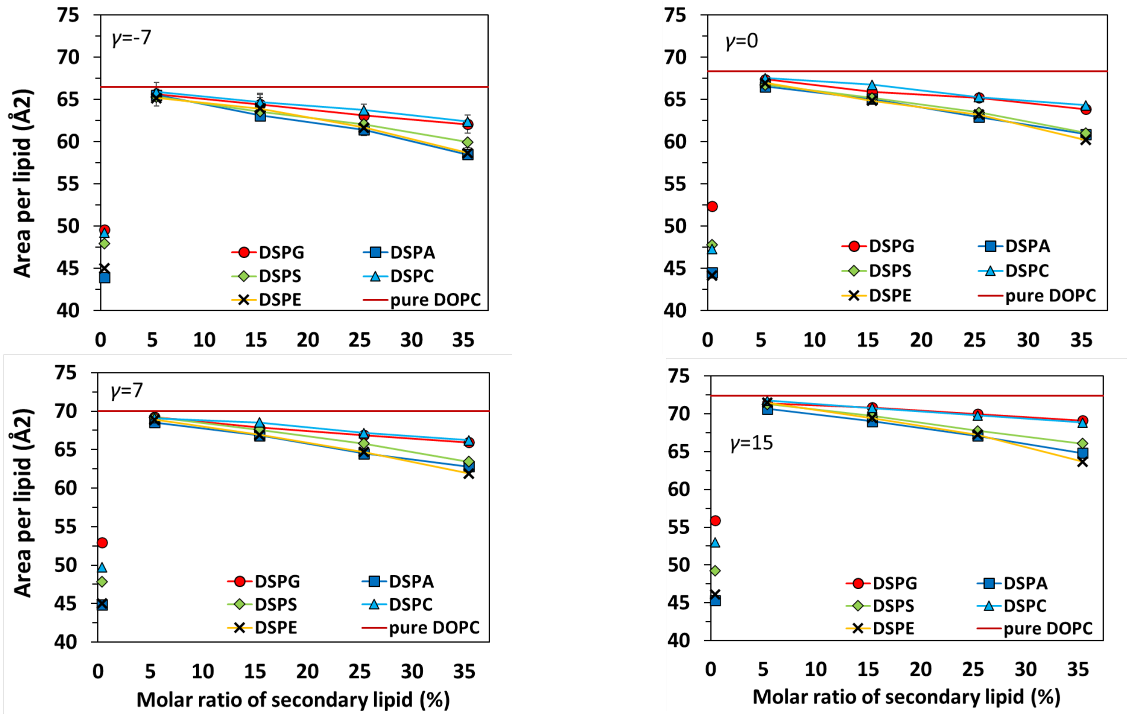

Figure S1. Area per lipid as a function of molar ratio of secondary lipids for all systems studied through all-atom simulations. The straight horizontal line in all plots represent the area per lipid values of pure DOPC. Leftmost symbols in each plot are area per lipid results for systems of pure secondary lipids; all of them have area per lipid values that are smaller than the values for pure DOPC.

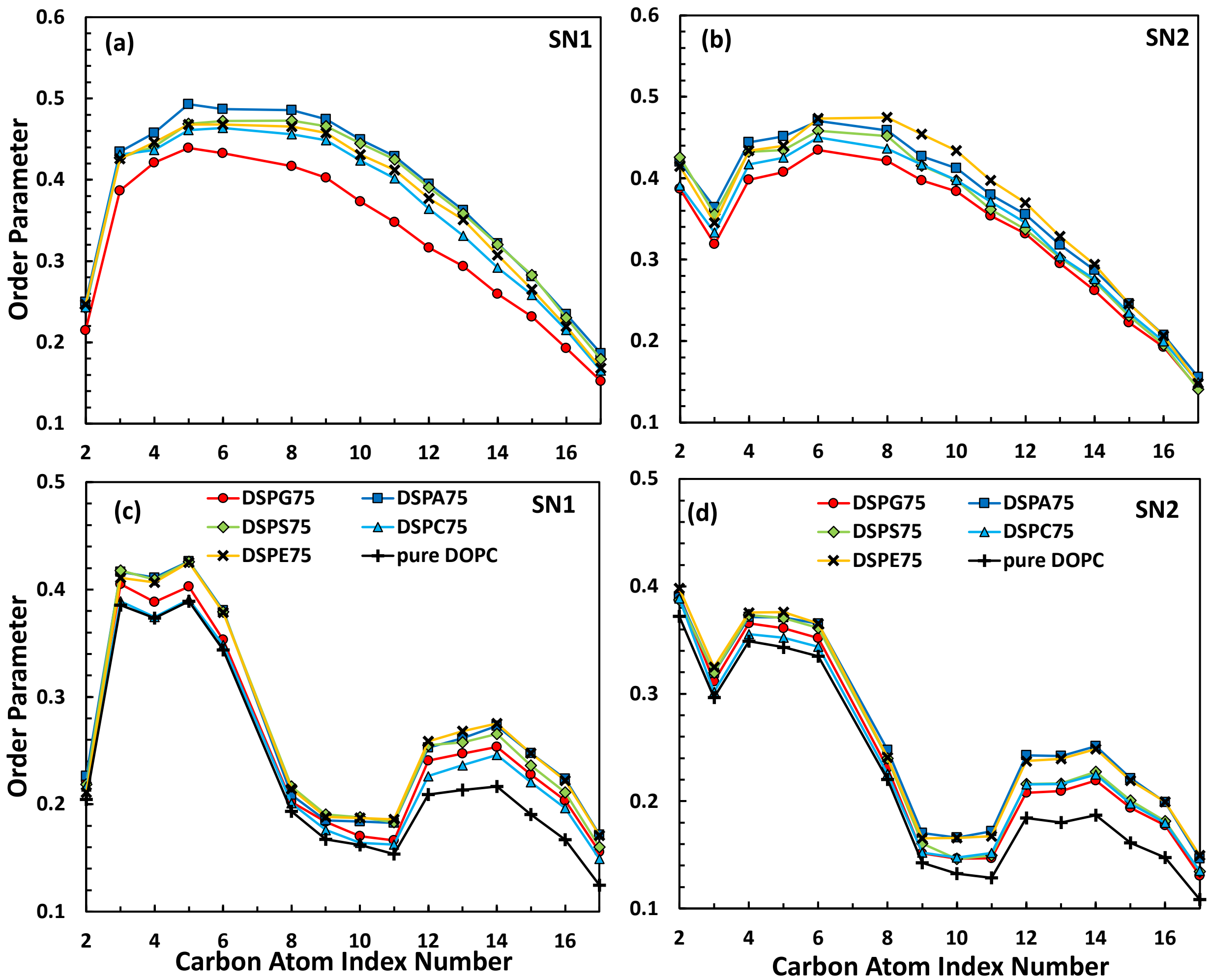

Figure S2. Order parameter S_C for the two lipid tails SN1 and SN2 in our 75:25 lipid bilayer mixtures at a surface tension $\gamma = 0$ mN/m, for the secondary lipids [(a) and (b)] and for DOPC [(c) and (d)]; results for a pure DOPC bilayer are also included in (c) and (d).

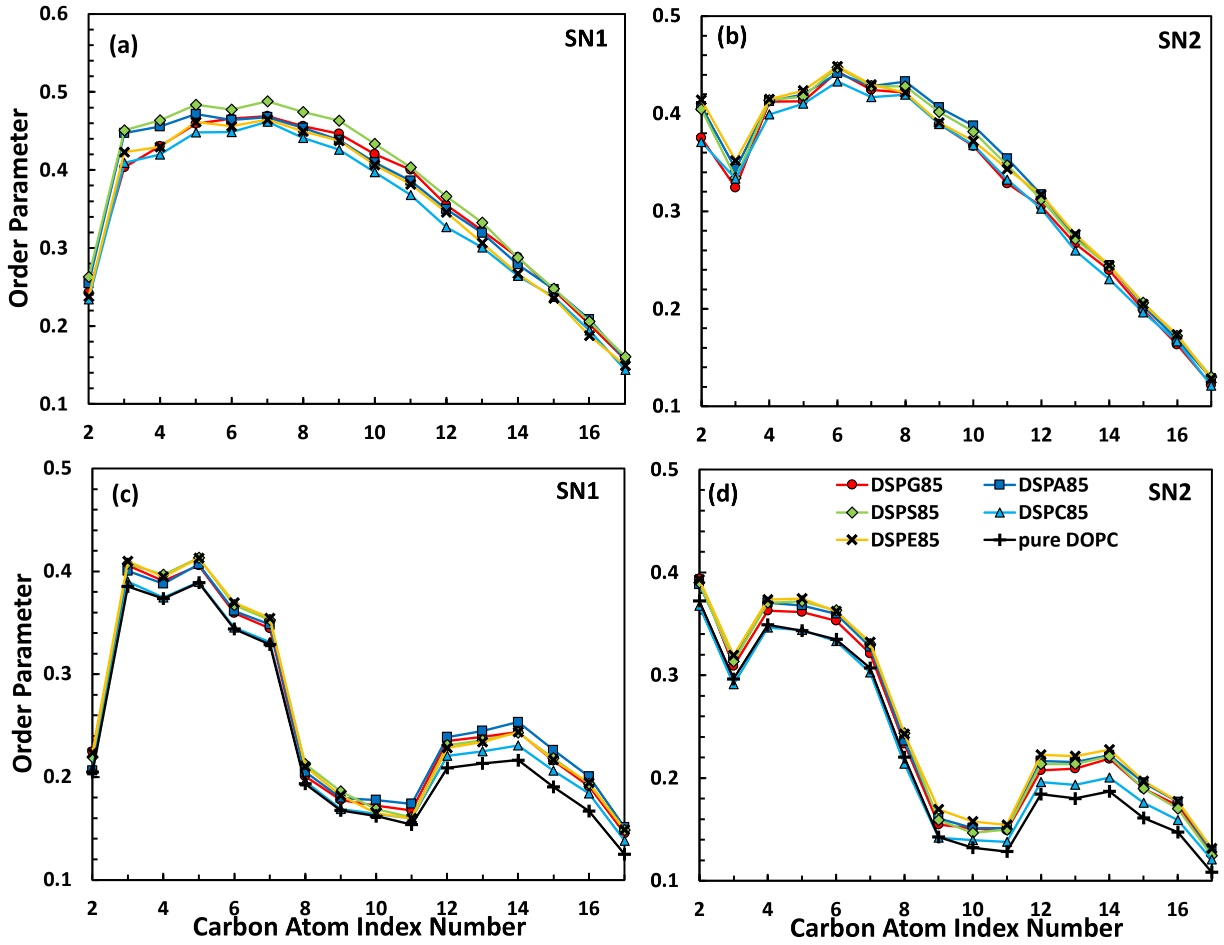

Figure S3. Order parameter S_C for the two lipid tails SN1 and SN2 in our 85:15 lipid bilayer mixtures at a surface tension $\gamma = 0$ mN/m, for the secondary lipids [(a) and (b)] and for DOPC [(c) and (d)]; results for a pure DOPC bilayer are also included in (c) and (d).

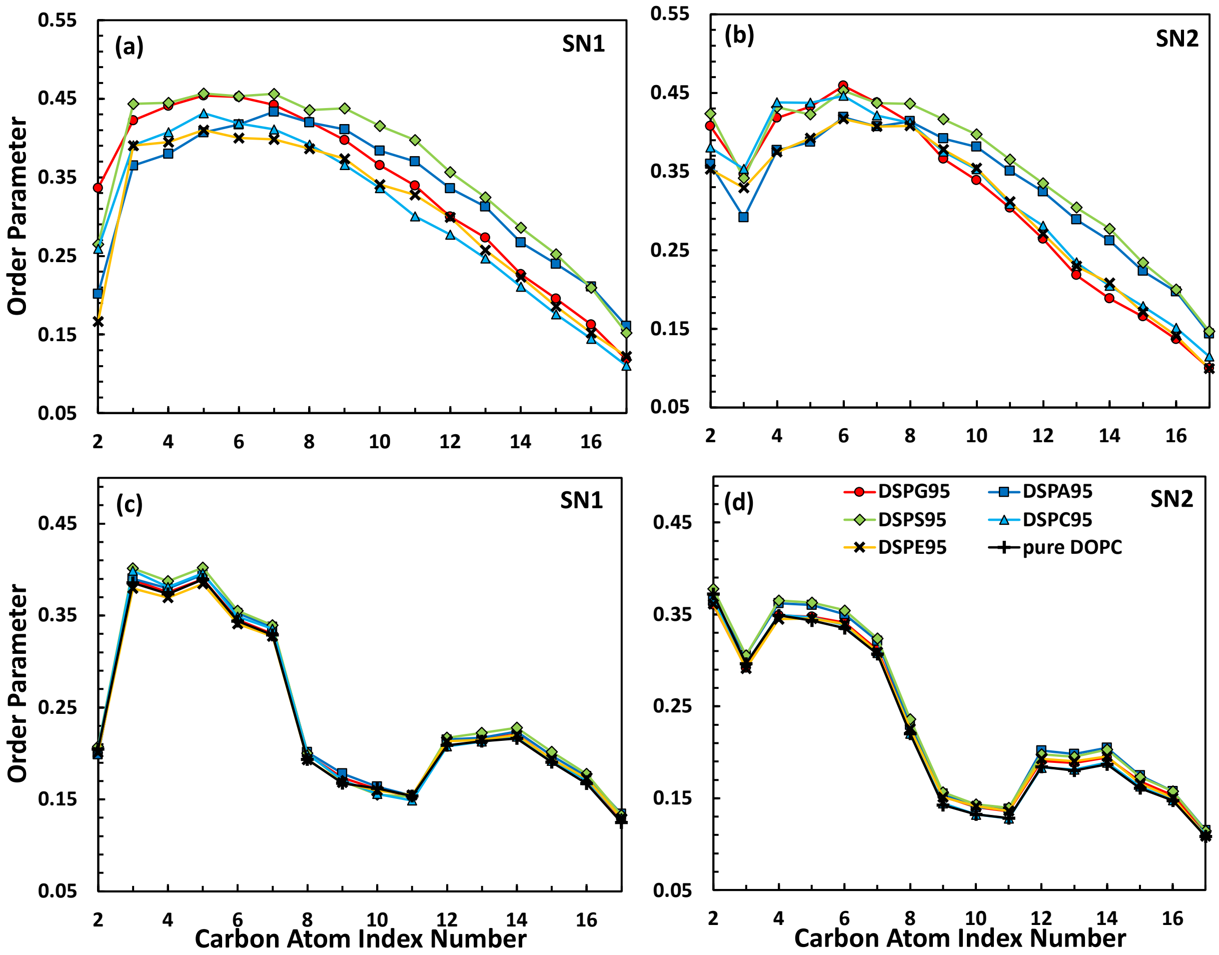

Figure S4. Order parameter S_C for the two lipid tails SN1 and SN2 in our 95:5 lipid bilayer mixtures at a surface tension $\gamma = 0$ mN/m, for the secondary lipids [(a) and (b)] and for DOPC [(c) and (d)]; results for a pure DOPC bilayer are also included in (c) and (d).

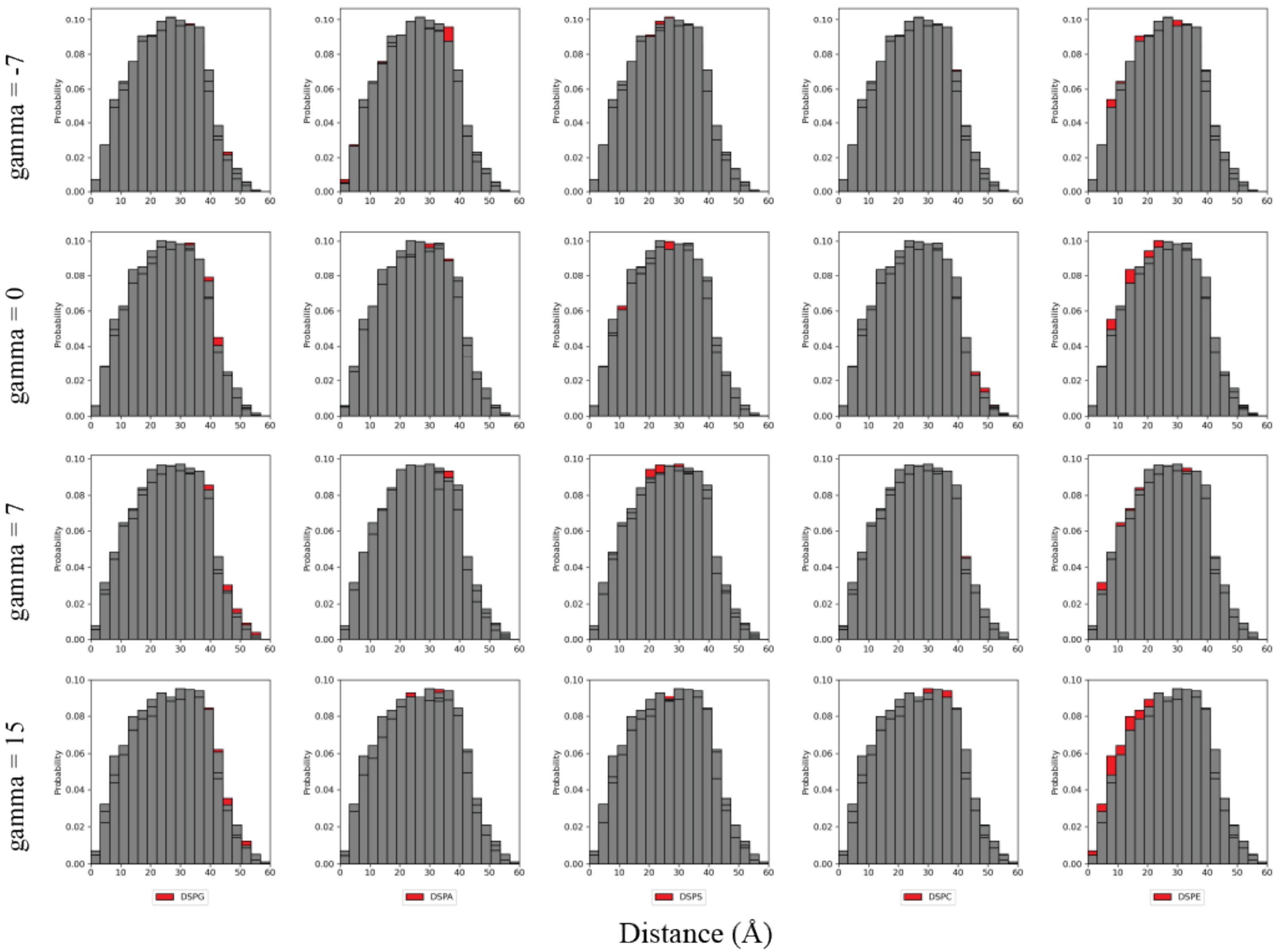

Figure S5. Probability histogram of distances of P-P atoms of secondary lipids for 65:35 mixtures at four different surface tensions. and how they compare against equivalent results for the other mixtures. For example, red bars in leftmost column illustrate the distances where the P-P distances between DSPG molecules in a mixture with DOPC have higher probabilities compared to equivalent results for DSPA, DSPS, DSPC and DSPE mixtures (in gray).

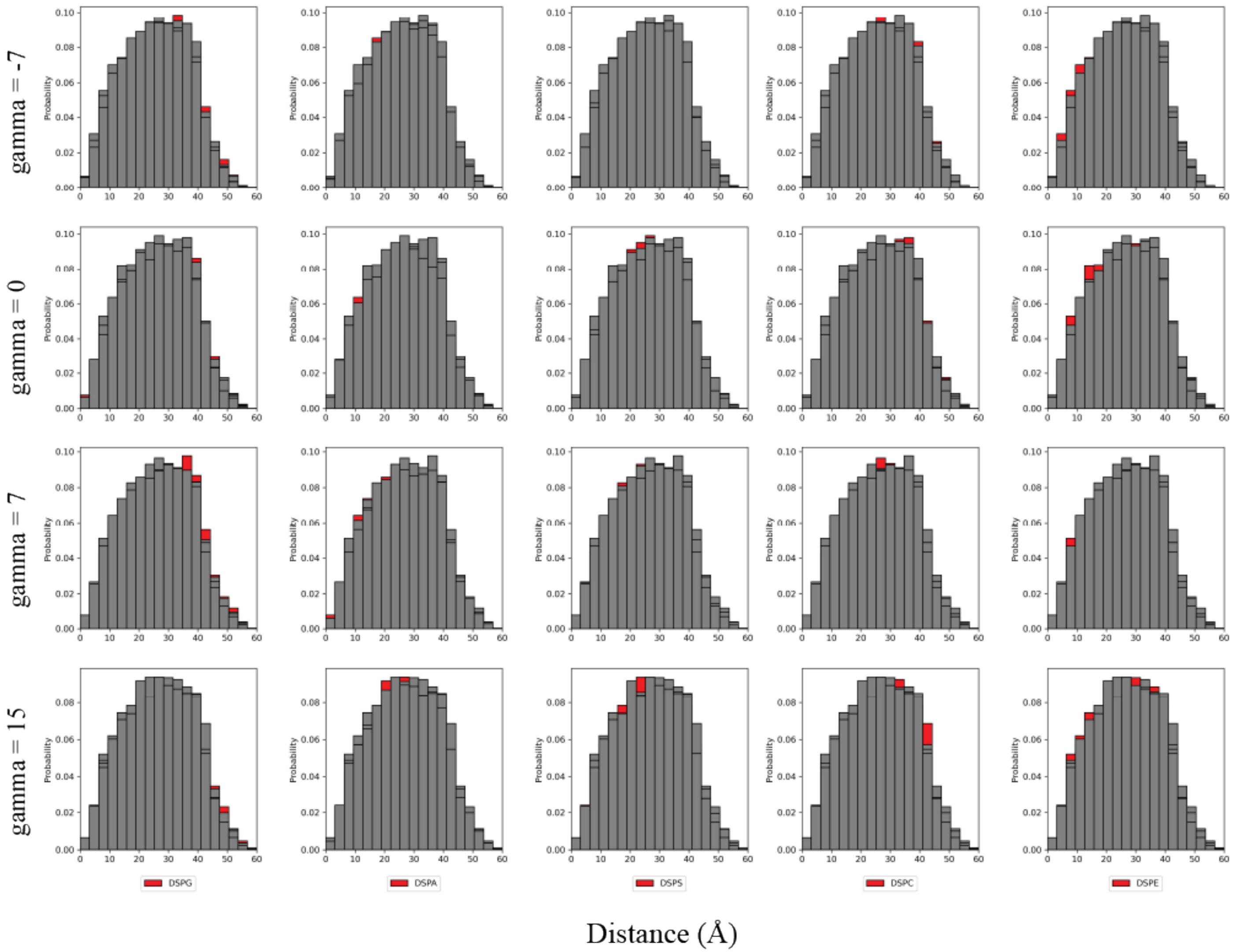

Figure S6. Probability histogram of distances of P-P atoms of secondary lipids for 75:25 mixtures at four different surface tensions. and how they compare against equivalent results for the other mixtures. For example, red bars in leftmost column illustrate the distances where the P-P distances between DSPG molecules in a mixture with DOPC have higher probabilities compared to equivalent results for DSPA, DSPS, DSPC and DSPE mixtures (in gray).

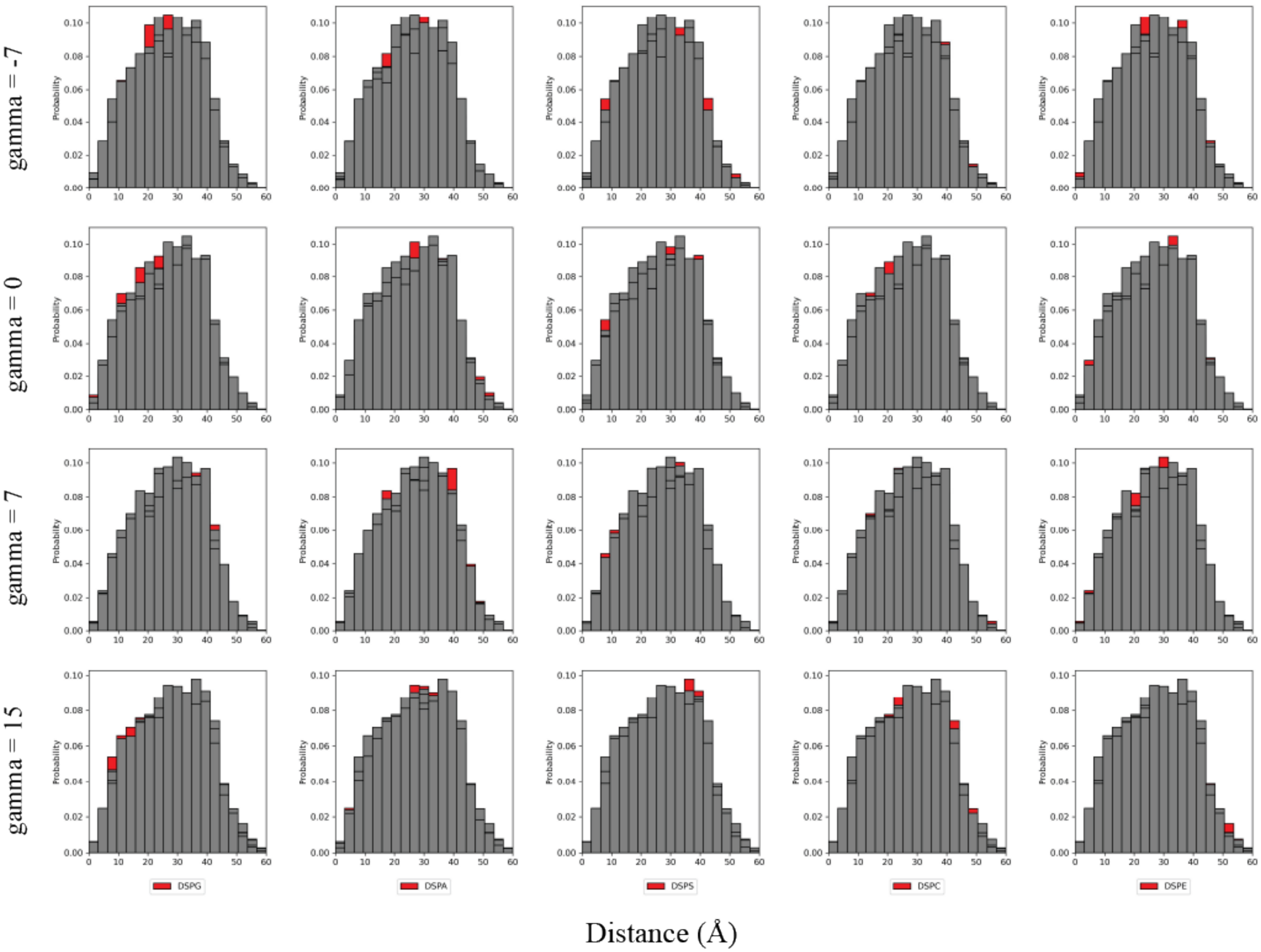

Figure S7. Probability histogram of distances of P-P atoms of secondary lipids for 85:15 mixtures at four different surface tensions. and how they compare against equivalent results for the other mixtures. For example, red bars in leftmost column illustrate the distances where the P-P distances between DSPG molecules in a mixture with DOPC have higher probabilities compared to equivalent results for DSPA, DSPS, DSPC and DSPE mixtures (in gray).

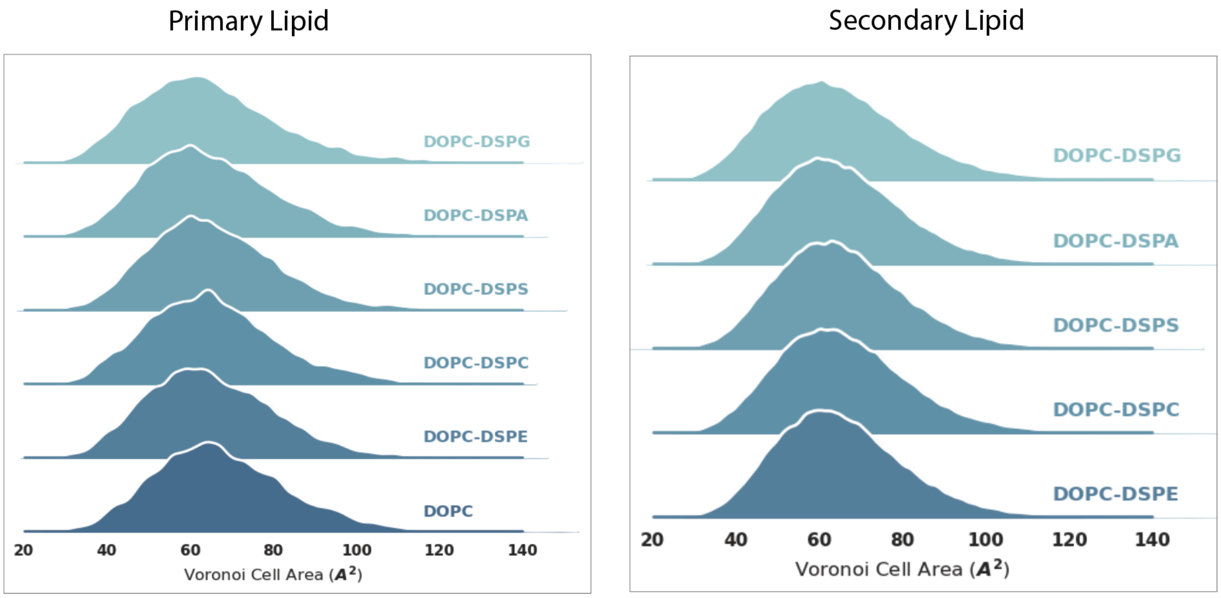

Figure S8. Histograms of Voronoi cell areas of (a) DOPC and (b) secondary lipids for 65:35 mixtures studied through coarse-grained simulations

References

(1) *Shaun, T. Student's t Table (Free Download) | Guide & Examples.* https://www.scribbr.com/statistics/students-t-table/ (accessed 2024-09-08).
